# Systematic reviews as a “lens of evidence”: Determinants of cost‐effectiveness of breast cancer screening

**DOI:** 10.1002/cam4.2498

**Published:** 2019-09-30

**Authors:** Olena Mandrik, Obinna Ikechukwu Ekwunife, Filip Meheus, Johan L. (Hans) Severens, Stefan Lhachimi, Carin A. Uyl‐de Groot, Raul Murillo

**Affiliations:** ^1^ Erasmus School of Health Policy & Management Erasmus University Rotterdam Rotterdam The Netherlands; ^2^ Health Economic and Decision Science (HEDS) School of Health and Related Research (ScHARR), The University of Sheffield Sheffield UK; ^3^ The section of Early Detection and Prevention International Agency for Research on Cancer Lyon France; ^4^ Collaborative Research Group for Evidence‐Based Public Health Department of Prevention and Evaluation Leibniz Institute for Prevention Research and Epidemiology BIPS/University of Bremen Bremen Germany; ^5^ Department of Clinical Pharmacy and Pharmacy Management Nnamdi Azikiwe University Awka Nigeria; ^6^ Institute for Medical Technology Assessment (iMTA) Erasmus University Rotterdam Rotterdam The Netherlands; ^7^ Institute for Public Health and Nursing Research—IPP Health Sciences Bremen University of Bremen Bremen Germany; ^8^ Centro Javeriano de Oncología Hospital Universitario San Ignacio Bogotá Colombia; ^9^ Faculty of Medicine Pontificia Universidad Javeriana Bogotá Colombia

**Keywords:** breast cancer screening, cost‐effectiveness, costs, review

## Abstract

Systematic reviews with economic components are important decision tools for stakeholders seeking to evaluate technologies, such as breast cancer screening (BCS) programs. This overview of systematic reviews explores the determinants of the cost‐effectiveness of BCS and assesses the quality of secondary evidence. The search identified 30 systematic reviews that reported on the determinants of the cost‐effectiveness of BCS, including the costs of breast cancer and BCS. While the quality of the reviews varied widely, only four out of 30 papers were considered to be of a high quality. We did not identify publication bias in the original evidence on the cost‐effectiveness of mammography screening; however, we highlight a need for improved clarity in both reporting and data verification. The reviews consisted mainly of studies from high‐income countries. Breast cancer costs varied widely among the studies. Factors leading to higher costs included: time (diagnosis and last months before death), later stage or metastases, recurrence of the disease, age below 64 years and type of follow‐up (more intensive or more specialized). Overall, screening with mammography was considered cost‐effective in the age range 50‐69 years in Western European and Northern American countries but not for older or younger women. Its cost‐effectiveness was questionable for low‐income settings and Asia. Mammography screening was more cost‐effective with biennial screening compared to annual screening and single reading using computer‐aided detection vs double reading. No information on the cost‐effectiveness of ultrasonography was found, and there is much uncertainty on the cost‐effectiveness of CBE because of methodological limitations.

## Graphical abstract

Multiple reviews report on the determinants of the cost‐effectiveness of breast cancer screening. However, only a few of these are of high quality. These reviews suggest that while the cost‐effectiveness of screening mammography is favorable in general, it is impacted by population (age, incidences), screening (interval and method), and setting (national income, geographic setting) characteristics. The cost‐effectiveness of ultrasonography and clinical breast examination is questionable. No risk of publication bias in the original evidence was identified, though high variability and uncertainty in the original and secondary evidence may limit the value of these reviews.

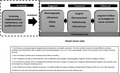

## 1 INTRODUCTION

Systematic reviews are widely accepted as a tool to increase the flow of scientific information.^1^ A dramatic increase in primary health economic studies has led to a consequent proliferation of systematic reviews synthesizing this economic evidence. These reviews may serve as a decision tool for stakeholders by evaluating the methodological rigor of the available economic evidence, defining principal cost drivers, summarizing variability in economic outcomes, or identifying the scientific gaps requiring further exploration. As such, these summaries are especially useful for the prospective evaluation of large‐scale programs requiring significant implementation and maintenance funding, such as breast cancer screening (BCS).

Breast cancer is the leading cause of death from cancer among women worldwide.^2^ Large randomized controlled trials and cohort studies, mainly conducted in North America and European countries, indicate that breast cancer mortality can be reduced by implementing screening mammography among women aged 50‐59 years.^3^ While there are multiple discussions on the benefit to harm ratio of screening mammography, it is generally considered as favorable by most of the systematic reviews synthesizing outcomes from randomized clinical trials and observational or population studies.^3^ Thus, guidelines from international cancer networks—including the European Union Council Recommendation on Cancer Screening—recommend mammography screening for this segment of the population.^4^, ^5^, ^6^ Scarce capacity limits application of these recommendations in low‐income settings, where clinical breast examination (CBE), breast self‐examination (BSE), and screening ultrasonography may be recommended by local guidelines either as individual or supplementary interventions.^7^, ^8^, ^9^, ^10^, ^11^, ^12^, ^13^, ^14^, ^15^

To define if a screening program provides value for money, cost‐effectiveness analysis is used. Cost‐effectiveness analysis is a comparative method, which combines relative costs and outcomes of different interventions into a single metric—the cost‐effectiveness ratio (CER) or incremental cost‐effectiveness ratio (ICER). The most frequently used outcome measures in cost‐effectiveness assessment of chronic illnesses, such as breast cancer, are quality‐adjusted life years (QALYs, a measure of disease burden including the quality and duration of life) or disability‐adjusted life years (DALYs, a measure of disease burden including the disability and years of life lost). Depending on the viewpoint of the stakeholders, cost‐effectiveness analysis can consider a variety of costs, such as direct costs (costs of screening, treatment, follow‐up, etc) and indirect costs (productivity loss for patient and caregiver). While multiple approaches to interpreting cost‐effectiveness exist,^16^ new technologies, in general, are considered to have a favorable CER if it is lower than the threshold established in the country or if it is less than average per capita income per DALY.^17^

The cost‐effectiveness of BCS programs is dependent on economic and healthcare system settings as well as the methodological approaches toward evaluation. The factors that would affect the cost‐effectiveness of BCS in populations would include patient characteristics and epidemiological factors, screening accuracy, coverage and screening uptake, access to diagnosis and treatment, and costs of both breast cancer and implementation of screening programs. While multiple reviews on economic evaluations around BCS have been published over the last decades, no research to our knowledge has summarized these reviews’ findings on the cost‐effectiveness of the screening programs. In the current overview, we aim to explore determinants of the cost‐effectiveness of BCS according to existing systematic reviews.

## 2 METHODS

The design of this study was reported in the published protocol, available open‐access online,^18^ and registered with the International Prospective Register of Systematic Reviews (PROSPERO), registration number CRD42016050765. We systematically searched PubMed via Medline, Scopus, Embase, and Cochrane databases in August 2016 and conducted updates and searches for gray literature in April 2018 and again in August 2018 (Appendix 1). As a deviation from the protocol, we included two systematic reviews that conducted the search in only one country—the USA, since they presented the most comprehensive data on the costs of breast cancer.

The quality of the included reviews was assessed by using the Assessing the Methodological Quality of Systematic Reviews (AMSTAR) checklist^19^ relevant for systematic reviews of cost and cost‐effectiveness outcomes and an additional question on transferability of the findings. We considered that the conclusions of the reviews were transferable to comparable jurisdictions, if the reviews reported low variability and uncertainty of the results, and considered the quality of original evidence as high or sufficient (Appendix 2 presents the decision framework). Furthermore, we narratively summarized the outcomes of the reviews that had a quality score of three or higher, considering the reviews with lower scores as nonsystematic. To explore the impact of the funding of the study, specialization of the department of the corresponding author, geographic focus of the review's search, year of the search, and the outcomes reported (cost or cost‐effectiveness), we used a stepwise multiple regression.

To analyze publication bias in the original evidence, we assessed the distribution of ICER per outcomes expressed in life years gained, QALYs, or DALYs in the reviews reporting on the cost‐effectiveness of screening mammography comparing to no screening. Similar to Bell et al (2006),^20^ we considered that publication bias exists if the published ratios cluster around the countries’ decision thresholds. Countries’ acceptability thresholds were expressed from one to three times the gross domestic product (GDP) per capita^21^ in the year prior to the publication year of the original evidence.

## 3 RESULTS

**Figure 1:**
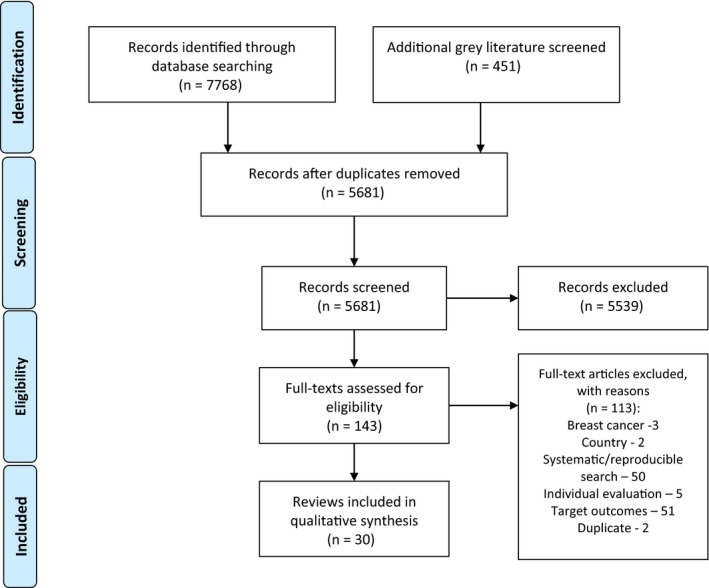
PRISMA 2009 Flow diagram

We identified 7768 abstracts through our database search, with 451 more gray literature sources reviewed (Figure 1). The interrater reliability between the two reviewers for decisions on full‐text inclusion was 92% (Cohen's kappa = 0.7; substantial agreement). The excluded reviews and reasons for the exclusion are outlined in Appendix 3.

**Figure 2:**
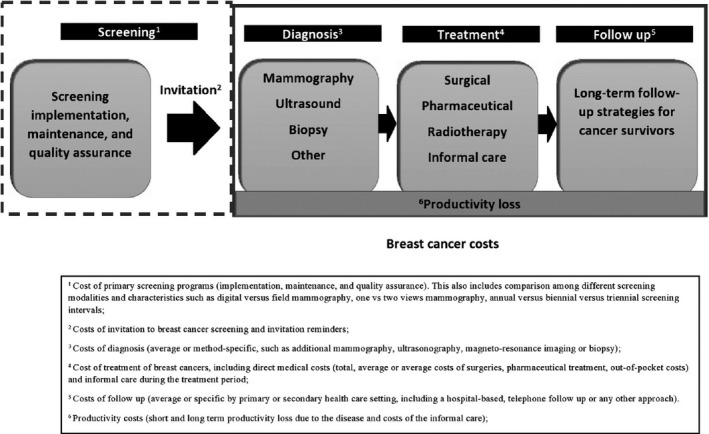
The conceptual framework of the review

Out of 30 included reviews, 14 reported data on the costs of breast cancer and 16 on the costs of BCS (the characteristics of the included studies are indicated in Appendix 4). Most reviews did not limit their search to a particular setting; some, however, aimed to identify studies comparable to either the UK,^22^, ^23^ North America,^24^, ^25^, ^26^, ^27^, ^28^, ^29^ Sweden,^30^ Iran,^31^ Asia,^32^ or high^33^‐ or low^34^‐income countries. Most studies included in the reviews were conducted in high‐income settings (Appendix 4), with little original research available for low‐and middle‐income countries. In particular, these reviews related to the cost‐effectiveness of BCS in Brazil, India, Mexico, Turkey, Ghana, and Egypt and productivity loss in Brazil, Peru, and Pakistan. In total, only two included reviews reported low variability and uncertainty in the synthesized results; the results of these reviews were considered applicable to high‐income countries.^35^, ^36^ The results of the studies are presented according to the described framework (Figure 2), reporting first the breast cancer costs and then the cost and cost‐effectiveness of BCS.

### 3.1 The determinants of breast cancer costs

From a macroperspective point of view, almost half of breast cancer costs are related to medication costs, with nonmedical costs and productivity costs taking a quarter each.^37^, ^38^ According to Foster et al,^39^ the financial impact of breast cancer (assessed on a macrolevel in the USA and Australia) was related to high trastuzumab costs and discard on its dispended prescriptions. The results from the five reviews concluded that direct medical breast cancer costs on a microlevel increased in the initial year after diagnosis and the last months before death (for out‐of‐pocket costs—the last 12 months of life^40^), later stage or metastatic breast cancer patients, receiving adjuvant chemotherapy, recurrence of the disease than the initial cancer, and age younger than 64 years (vs an older age).^37^, ^39^, ^40^, ^41^

Six reviews, mainly based on the same original evidence, reported resource use or costs of breast cancer follow‐up (Appendix 5).^22^, ^30^, ^35^, ^42^, ^43^, ^44^ The highest rate of resource use was for follow‐up visits and follow‐up mammography,^44^ while the frequency of visits decreased twice in the initial 4 years after treatment.^43^ The follow‐up costs could be affected by poor continuity of the doctor‐patient relationship, with patients seeing multiple doctors during the follow‐up and doing almost twice the recommended number of visits.^43^ The cost of intensive follow‐up was 2‐5 times higher than minimal follow‐up while not having an impact on survival 22, 35, 42, 43; the cost of follow‐up was lower if it was in the primary vs secondary setting,^22^, ^30^, ^35^, ^43^ nurse‐led,^44^ or nurse‐led phone follow‐up combined with an educational program,^30^ or phone or through mobile application technologies.^22^, ^35^ Even though no impact on clinical outcomes was recorded with follow‐up in the primary setting, patients’ satisfaction was much higher with specialist follow‐up.^22^, ^30^

Seven reviews concluded that breast cancer has a significant impact on the productivity of women in all the countries considered,^24^, ^36^, ^38^, ^39^, ^40^, ^41^, ^45^ affecting the unemployment rate of breast cancer survivors and causing financial hardship for families of cancer patients.^40^ The unemployment rate varied widely among the studies and the countries (from 20%‐55% at 3‐24 months in Germany, the USA, and France to 12%‐43% after 6‐9 years in the USA, Sweden, Canada, and Germany). Because of heterogeneity in methods, background unemployment rates, and population characteristics, it is impossible to conclude on any real differences in the geographic settings.^24^, ^36^, ^38^, ^40^, ^45^ Similarly, the average return to work varied widely among the countries, for instance, being three times longer in the Netherlands than in Sweden.^38^

### 3.2 Determinants of breast cancer screening costs and cost‐effectiveness

Only one narrative review reported the costs of organized invitations, with the cost of follow‐up reminder being 3‐9 times higher than the cost of initial invitation.^25^ In general, mammography was considered to be cost‐effective to screen 50‐ to 69‐year‐old women in three reviews reporting studies from Western European and North American countries,^31^, ^32^ although questionable for low‐income settings^34^ and Asian regions (that was argued by differences in incidence rates and density of breast tissues)^32^ (Table 1). BCS costs were lower with biennial mammography compared with annual mammography in the review of Health Quality Ontario (2016)^29^; consequently, biennial mammography screening was considered to be the most cost‐effective option in the review by Rashidian et al (2013), although the range of cost per life year gained was the lowest for triennial screening.^31^ Incremental cost per life year gained of continuing mammography screening for women above 65 years of age compared with stopping regular screening at that age was 34000‐88000 USD (2002). Although it could possibly be lower if screening decision would be based on women's health state.^27^ The cost‐effectiveness of screening of women younger than 50 years of age had a range within the recommendations of the World Health Organization of three times GDP per capita (14000‐26200 USD per life year gained in the USA in 1994 and 45000 USD per QALY gained in the UK, 2010). However, the authors of this systematic review did not consider BCS for this group of women cost‐effective.^31^

**Table 1:** Breast cancer screening cost and cost‐effectiveness outcomes

Author, year	Searched outcomes	Reported outcomes	Reported conclusions on cost‐effectiveness or heterogeneity
Wagner, 1998^25^	Costs of invitation for MM (USA, Australia) (a) Unit costs (b) Cost per woman screened (c) Cost of follow‐up reminders	(a) 0.45‐2.78 USD (b) 0.96‐5.88 USD (c) 3.25‐26.81 USD	More research is needed to assess the cost‐effectiveness of patient reminders
Baxter, 2001^26^	Cost of BSE education programs per competent frequent self‐examiner added	574‐848 USD (USA, 1993)	No conclusion
Dinnes, 2001^23^	(a) Incremental cost per additional cancer detected (UK, France, USA)	(a) 1162‐2221 GBP, 21838FF, 25523 USD	Cost‐effectiveness estimates have been produced which lie within the range of what may be considered to be “cost‐effective”.
Ho, 2002^28^	(a) Resource use with DM vs FSM (1) Examination time (2) Repeat examinations (b) Incremental capital equipment (1995‐2001, USD) (c) Annual operating costs	(a) Resource use with DM vs FSM (1) < by 5.3‐6.3 min (2) <1.48%‐6% (b) 50000‐284000 USD (c) Not consistent	DM equipment is more expensive than FSM, but has reduced time and reduced repeats
Mandelblatt, 2003^27^	Cost and cost‐effectiveness extending BCS above 65 y (a) Diagnosis costs (2002, USD) (b) Treatment costs (2002, USD) (c) Incremental costs per life year saved	(a) 451‐2520 USD (b) 7991 (surgery only)‐45220 USD 66‐194 USD (c) 34000‐88000 USD	Health state of women (risk of complications), age
Baron, 2010^33^	Cost of reminders per additional MM for those appearing on time vs requiring additional prompting	75 USD vs 118 USD	Patients’ punctuality impacts the costs
Baron, 2008^49^	Economic efficiency of reducing structural barriers in increasing breast cancer screening	No studies were found	Not applicable
Rashidian, 2013^31^	Cost‐effectiveness of MM screening (a) Cost per life year, mixed age (b) CER for 50‐ to 70‐year‐old (1) Cost per LYG, biennial (2) Cost per LYG, annual (3) Cost per LYG, triennial (4) Cost per QALY (all intervals) (5) Cost per DALY (1 study) (6) Cost per cancer detected (c) CER for women over 70 (1) Cost per LYG, annual (2) Cost per QALY (d) CER for women younger 50 (1) Cost per LYG (2) Cost per QALY	(a) 1634 USD ( India)‐64400 USD (Australia) (b) CER for 50‐ to 70‐year‐old (1) 2685 USD (UK, 1993)‐21400 USD (USA, 1997) (2) 15500 USD (USA, 1994)‐45700 (USA, 1997) (3) 4343USD (UK, 1998)—13081 (Australia, 1993) (4) 9801 USD (Slovenia, 2008‐46500 (USA, 1997) (5) 75 (Africa)—915 USD (North America, 2006) (6) 8424USD (Spain, 1996)‐17202 USD (Norway, 1999) (c) BCS MM for women over 70 (1) 35000 USD (USA, 1994) (2) 8119*‐*27751 USD (other review) (d) CER for women younger 50 (1) 14000*−*26200 USD (USA, 1994) (2) 44692 (UK, 2010)	Biennial screening test for those aged 50‐70 y seems to be the most cost‐effective option. Screening those aged less than 50 is not recommended.
Yoo, 2013^32^	Cost‐effectiveness of MM BCS in Western and Asian countries (a) Cost per LYG or QALY (Asian countries) (b) Cost per LYG or QALY (Western Europe) (c) Logged CE/per capita GDP ratio predictions	(a) 3308 USD (India, 2008) −90771 USD (China, 2007) (b) 3235 USD (NL, 1991)‐48884 USD (USA, 2011) (c) −0.69 (Spain, 2011)‐1.69 (China, 2007)	Incidence rate and racial characteristics (breast tissue density) affect the outcome. Cost‐effective cutoff point of breast cancer incidence rate was 45.04; it exactly divided countries into Western and Asian countries.
Zelle, 2013^34^	Cost‐effectiveness of BCS alternatives (a) MM (b) CBE (c) Other BCS considered cost‐effective	(a) Cost‐effective: sub‐Saharan Africa and South East Asia (2248‐4596 USD/DALY), Mexico (22000 ID/DALY), Poland, Turkey (2006, 2011), not rational—Iran, not cost‐effective—Ghana. (b) Cost‐effective: India, Ghana (1299 USD/DALY), Egypt (c) Tactile imaging (incremental costs not reported)	BCS may be economically attractive in LMICs—yet there is little evidence to provide specific recommendations on screening by MM vs CBE, the frequency of screening, or the target population.
Koleva‐Kolarova, 2015^48^	Cost per outcome (undefined) in nonconverted currency	1800 GBP (UK, 1993) −715000 EUR (Spain, 2011)	Most reported screening regimens fulfilled the WHO criteria with the exception of some very intensive USA, Spanish and Indian scenarios.
Li, 2015^50^	(a) Cost per LYG with MM screening (India 2008, Brazil 2012) (b) Cost‐effectiveness of CBE vs MM (India) (c) CAD vs double reading (2015)	(a) 3468 USD, 6516 USD (b) Cost‐effective (no ICER reported) (c) Cost‐effective (no ICER reported)	Results from high‐income countries are not applicable to low‐income settings and should be accessed on individual basis
Abdel‐Aleem, 2016^51^	Total costs per screened patient (USA, 2009): (a) Stationary full digital screening unit (b) Mobile full digital screening unit (c) Mobile film screening unit	(a) 41 USD (b) 102 USD (c) 86 USD	The cost of screening per woman may be higher for mobile clinics than for permanent clinics (low certainty)
Health Quality Ontario, 2016^29^	BCS costs per 1000 women (1 study): (a) Biennial in 50‐74 y.o. (1) MM (2) MM + US, dense breast, (+incremental LYG and QALY) (3) MM + US, heterogeneously or dense breast (b) Annual 40‐74 (1) MM (2) MM + US dense breast (+incremental LYG and QALY) (3) MM + US, heterogeneously or dense breast	(a) Biennial in 50*‐*74 y.o. (1) 3.02 mln USD (2) 3.08 mln USD (1.2 LYG, 1.1 QALY) (3) 3.39 mln USD (2.1 LYG, 1.7 QALY) (b) Annual 40‐74 (1) 5.15 mln USD (2) 5.42 mln USD (3.6 LYG, 3.1 QALY) (3) 6.58 mln USD (3.7 LYG, 3.0 QALY)	No studies on MM + US to screen average‐risk women
Arnold, 2017^42^	Personalized screening (screening interval is dependent on personal risk), general population (a) cost/QALY (2014) (b) Difference between lower and higher risk women (2014) (c) Cost of screening, low risk (d) Cost of screening, average risk (e) Cost of screening, moderate risk	(a) Dominant‐246000 USD (USA) (b) 2000‐2500 USD (c) 247‐2840USD (d) 377‐1656USD (e) 1248‐5304USD	Lower risk women have lower screening cots
Posso, 2017^47^	(a) Incremental cost of double vs single reading (2005, PPP) (b) Cost per LYG of single reading + CAD vs double reading (2015) (c) Cost per cancer detected of double reading vs single (2015)	(a) 25.7 USD‐271886 USD (b) 2951USD (c) 24717 USD	Double reading was not cost‐effective in comparison to single reading or single reading + CAD

Abbreviation: BCS, breast cancer screening; BSE, breast self‐examination; CAD, Computer‐Aided Detection; CER, cost‐effectiveness ratio; CPI, consumer price index for medical care; DALY, disability adjusted life years; DM, digital mammography; EUR, Euro; FSM, film screening mammography; GBP, Great British Pound; GDP, gross domestic product; ID, international dollars; LYG, life years gained; MM, mammography; NL, the Netherlands; PPP, Purchasing power‐parity; UK, united kingdom; US, ultrasonography; USD, United States dollar; QALY, quality adjusted life years; y.o., years old.

Several reviews assessed the impact of different organizational aspects on the cost‐effectiveness of screening mammography. The review of Baron (2010) identified more than a 50% increase in cost of invitation for those women requiring a follow‐up reminder to come for screening.^33^ The review by Ho et al (2002) identified significantly higher costs of capital equipment for digital mammography compared to film mammography, although the former reduced the number of examination repeats (1.5%‐6%) and decreased examination time by around 5 minutes.^28^ Double reading was considered not to be a cost‐effective intervention in comparison to single reading with computer‐aided detection in the review of Posso et al (2017).^47^ Another review estimated the costs of personalizing screening intervals considering an individual's cancer risk, concluding higher cost (2000‐2500 USD, 2014) for a higher risk population.^42^

While mammography was a target intervention in most of the reviews, three publications reported BCS costs for the other screening approaches (Table 1). Zelle et al (2013) reported that CBE can be a cost‐effective screening method for some low‐income settings (India, Ghana, and Egypt).^34^ Baxter et al (2001) estimated the range of costs (574‐848 USD) necessary to educate one woman to regularly and competently practice breast self‐examination.^26^ The report by Health Quality Ontario targeted to identify the cost‐effectiveness of adjunct ultrasonography including women of general risk; no study for average risk women reporting the cost‐effectiveness of combined screening was identified though.^29^

### 3.3 Quality and bias in evidence on cost‐effectiveness of breast cancer screening

The quality of the included reviews ranged from 1 to 7 (from a maximum possible of 9 in AMSTAR), of which 25 reviews had a quality score of three or above and were included in the data synthesis (Table 2).

**Table 2:**
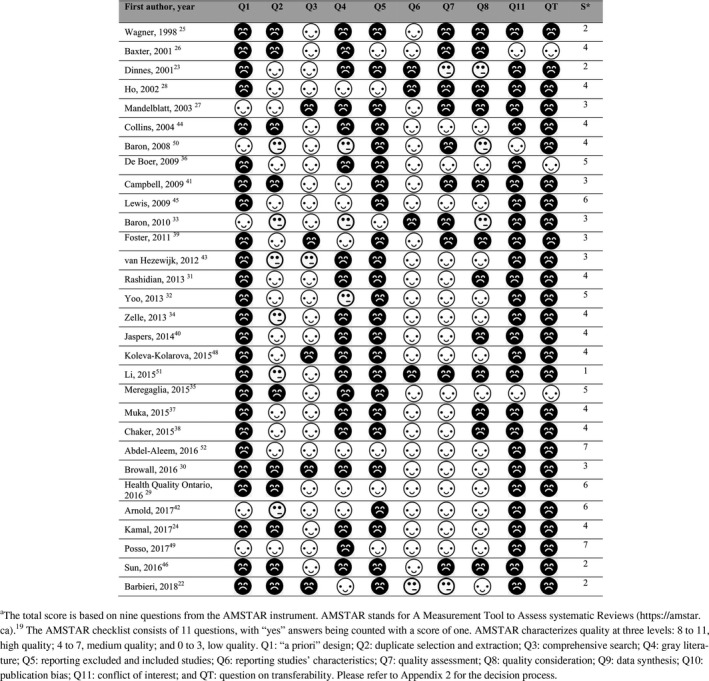
Assessment of quality of included systematic reviews (Score 9 is the maximum)

**Figure 3:**
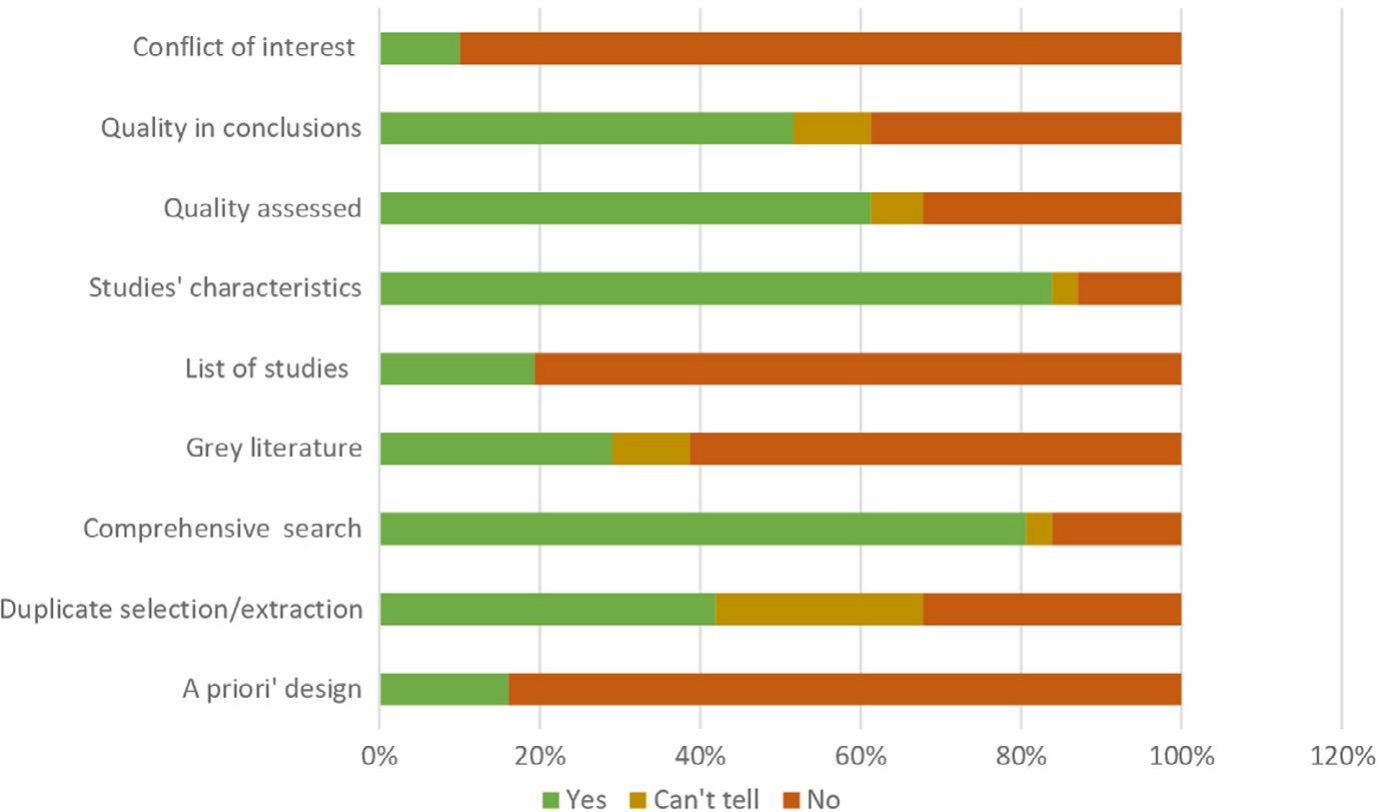
Quality of the included systematic reviews

From the relevant AMSTAR criteria, most systematic reviews had a comprehensive literature search (81%) and reported study characteristics (84%). Only three reviews (10%) reported the conflict of interest for included original research, and only five (17%) reported having a protocol (Figure 3). A stepwise multiple regression model evaluated the impact of factors on the AMSTAR score. The final regression model excluded factors such as funding of the study and the specialization of the department of the corresponding author, including a geographic focus of the review's search (world vs country or region‐specific, *P* < .05), year of the search (*P* < .1), and reporting cost‐effectiveness parameters vs only cost parameters (*P* = .174). The model predicted 47% of variance in the AMSTAR score with residual standard error of 1.133 on 23 degrees of freedom.

Four systematic reviews on the cost‐effectiveness of BCS in comparison to no screening reported the outcomes from 67 studies in total (Appendix 6). While most of the inclusion criteria were similar among the three reviews, 13%‐60% of the original articles were not included in one or another review when expected. Among those studies included in two or more reviews, 10 out of 22 (45%) had different ranges for reported ICERs; these differences in the reporting were not related to a particular trend of cost‐effectiveness estimates. Two of these four systematic reviews were used to assess the risk of publication bias. The other two were excluded due to poor reporting of programs and outcomes. We did not identify a risk of publication bias in the original studies with differences between GDP and ICER varying widely depending on the country of evaluation, screening interval, and age groups (Appendix 6).

## 4 DISCUSSION

This systematic review assessed the determinants of the cost‐effectiveness of BCS as well as the methods and quality of 30 included systematic reviews.

### 4.1 Determinants of the cost‐effectiveness of mammography screening

The determinants of the cost‐effectiveness of BCS were split into two stages indicated in the conceptual framework, the screening costs, and the breast cancer costs, with the later including the costs related to the diagnosis, treatment, and follow‐up. Breast cancer costs were affected by the disease characteristics (eg, stage and incidence), patients’ characteristics (eg, age), health provider characteristics (eg, nurse vs general practitioner follow‐up), and health system characteristics (eg, discard of dispensed prescriptions and lack of societal insurance).^22^, ^30^, ^35^, ^37^, ^39^, ^40^, ^41^, ^42^, ^43^, ^44^, ^45^, ^47^ There was not enough evidence to evaluate how the impact of these factors on breast cancer cost would differ between high‐ and low‐income countries. While low‐income countries have less financial means and lower breast cancer expenses, wastage and improper resource allocation also contribute to the inefficiency of the healthcare systems in these jurisdictions.^52^ With high breast cancer mortality rates in countries with limited resources,^52^, ^53^ one may speculate that improvement of the efficiency in healthcare systems,^52^ including cancer treatment in national health insurance coverage,^54^ gaining capacity, and setting early cancer detection programs,^55^ should go prior to BCS implementation.

The cost and cost‐effectiveness outcomes related to screening were influenced by population characteristics (eg, age, personal cancer risk, and breast tissue density), screening organization (eg, screening interval, prompting, mammography type, and number of readers), and disease characteristics (eg, breast cancer incidence).^27^, ^31^, ^32^, ^33^, ^47^ Screening mammography was the most reported intervention in the reviews of the cost‐effectiveness of BCS and was generally accepted as cost‐effective for 50‐ to 69‐year‐old women in high‐income settings but not in low‐income settings or in Asian populations. What undervalues the economic assessments of BCS among younger and older women is that the evidence on the benefit/harm ratio of screening mammography in these populations is inconclusive or limited.^3^ Nevertheless, the conclusions on the cost‐effectiveness of screening mammography among women younger than 50 years old or older than 69 years old could vary from those presented if other clinical outcomes were selected, since the incidence of breast cancer (and so advanced cases prevented) was higher among the older population (while the younger population accumulated more life years gained) or if the individual‐based screening approach would be evaluated.

The results of this review have a dual‐fold impact. Firstly, policy makers should take the factors listed above seriously, especially when new screening programs are designed. These factors are prerequisites for an optimal implementation of a cost‐effective screening program, even though the BCS of 50‐ to 69 year‐old‐women is considered to be, in general, a cost‐effective intervention. A comprehensive cost‐effectiveness evaluation of BCS with long‐term forecasted outcomes supported by the evidence from local pilots would support efficient program design and functioning.

Secondly, the differences in healthcare systems, health providers, and populations as well as breast cancer costs and their determinants should be considered in estimations of the transferability of findings on the cost‐effectiveness of BCS. The transferability approaches suggest that, at a minimum, practice patterns and unit costs from the jurisdiction of interest should be considered.^56^ In relation to prevention approaches, not only the unit costs of the preventive intervention but also cost of the disease itself, would define transferability of cost‐effectiveness estimates. Considering the long‐term effect of the indirect costs related to breast cancer as well as high heterogeneity in cost outcomes by patient characteristics, an individual‐level approach to model‐based economic evaluations would be more relevant to capture the long‐term impact of the disease on both the well‐being and patient‐related costs. Finally, the model parametrization approaches, in particular, calibration, will also affect the transferability of the cost‐effectiveness results. Calibration is a strategy for quantifying unobserved model parameters to mimic the observed historical data. Considering that the natural history of the disease frequently includes undetected states and so transitions, calibration is frequently applied in health economic modeling approaches. The source of target data to fit in the calibration (whether it is a global, regional, or local source of statistics), would anchor the results narrowly (to one specific program or environment) or broadly (to multiple settings).

### 4.2 Other breast cancer screening modalities

Besides mammography, limited (for BSE and CBE) to no (for ultrasonography) information was reported on other screening modalities. Even though CBE and BSE are generally perceived as low‐investment approaches, they also require launch and maintenance costs related to the education and enrollment of women. Costs required for BSE education were reported in one review,^26^ and the cost‐effectiveness of CBE in another review, suggesting it to be a cost‐effective method for some low‐income settings (India, Ghana, and Egypt).^34^ The secondary sources are consistent in presenting sufficient evidence of no benefits but harms of BSE and on insufficient evidence of a mortality decrease in breast cancer with regular CBE.^3^ By this, models assessing the benefits of CBE would rely on intermediary outcomes—stage shifting of breast cancer—rather than real‐life data on mortality decrease, which underpins assessments of the cost‐effectiveness of this intervention.

### 4.3 Quality and bias in the evidence

While the quality of the reviews varied widely, only four of them were considered to be of a high quality (scored 6 or above on AMSTAR). Meanwhile, there was no clear relation between the quality of the included systematic reviews and their conclusions. Reviews having a wide geographic focus (world rather than targeting a certain country or region) and more recent search tend to have higher AMSTAR scores. Our more in‐depth analysis of reporting from four systematic reviews has shown potential risk of search, selection, extraction, and reporting mistakes in the reviews rather than biases in these studies. Similarly, we did not identify publication biases in the original evidence. We consider that cost‐effectiveness analyses of public preventive programs, such as BCS, may be at less risk of publication bias than pharmaceutical treatments. To improve the quality, reliability, and applicability of systematic reviews, the reviewers should refer to the developed guidelines in their methods, provide more transparent reporting of programs and outcomes, and consider the transferability of their findings.

### 4.4 Research and information gaps

Our overview shows that more original trial‐based economic evaluations along with pilots of CBE and ultrasonography with evidence on clinical and economic benefits are required. In addition, more high‐quality field‐based studies and reviews on the cost‐effectiveness of mammography screening in low‐ and middle‐income countries as well as the cost‐effectiveness of mammography screening among older women are also required. Economic evaluations of BCS considering personal risk stratification would be an asset for health decision‐making. A better understanding of costs related to informal care and indirect screening program costs would help to decide how these finances should be considered in economic analyses of breast cancer. Standard and better structured collection and reporting of costs would improve comparability among the studies. The risk of bias tool designed for the reviews reporting cost outcomes would help to interpret the results of these studies and potentially simplify their use in healthcare decision making.

### 4.5 Limitation

With the large scope of the searched literature, it is possible that we missed some of the important information, despite the comprehensive approach applied in this review. We also deviated from the protocol, including two reviews on which agreement between two raters was not reached. The AMSTAR tool was not fully applicable to assess the quality of the systematic reviews with cost and cost‐effectiveness outcomes. In addition, the applied transferability metric was not validated, and the use of a standard validated tool specific for systematic reviews of economic evaluations would be a preferable approach.

## 5 CONCLUSIONS

Screening mammography may be a potentially cost‐effective intervention, although how cost‐effective it is would depend on population characteristics (such as incidence and starting age for screening) and screening organization (screening interval and screening approach) as well as the direct and indirect costs of breast cancer and their determinants. No information on the determinants for the cost‐effectiveness of ultrasonography was retrieved, and the cost‐effectiveness of CBE is not certain because of methodological limitations. No risk of publication bias in the original evidence was identified, although high variability and uncertainty in both the original and secondary evidence may limit the value of these reviews.

## References

[1] Murad MH , Asi N , Alsawas M , Alahdab F . New evidence pyramid. Evid Based Med. 2016;21:125‐127. 27339128 10.1136/ebmed-2016-110401 PMC4975798

[2] WHO . Guidelines approved by the guidelines review committee WHO position paper on mammography screening. Geneva: World Health Organization; 2014. 25642524

[3] Mandrik O , Zielonke N , Meheus F , et al. Systematic reviews as a ‘lens of evidence’: determinants of benefits and harms of breast cancer screening. Int J Cancer. 2019;145(4):994‐1006. 30762235 10.1002/ijc.32211 PMC6619055

[4] Oeffinger KC , Fontham E , Etzioni R , et al. Breast cancer screening for women at average risk: 2015 Guideline update from the American Cancer Society. JAMA. 2015;314:1599‐1614. 26501536 10.1001/jama.2015.12783 PMC4831582

[5] Senkus E , Kyriakides S , Ohno S , et al. Primary breast cancer: ESMO clinical practice guidelines for diagnosis, treatment and follow‐up. Ann Oncol. 2015;26(Suppl 5):v8‐30. 26314782 10.1093/annonc/mdv298

[6] Nelson HD , Cantor A , Humphrey L , et al. Preventive services task force evidence syntheses, formerly systematic evidence reviews screening for breast cancer: a systematic review to update the 2009 US Preventive Services Task Force Recommendation. Rockville, MD: Agency for Healthcare Research and Quality (US); 2016. 26889531

[7] Protocol on Public Health Surveillance . Cancer of the breast and cervix [in Spanish]. National Institute of Health of Colombia;2016;1‐39. Version 02.

[8] Unified clinical protocol of the primary, secondary and tertiary medical help. Breast Cancer. Approved by the order #396 on 30.06.2015. The Ministry of Health of Ukraine; 2015.

[9] Martínez AO , González Martín A , Rodríguez Monteagudo RL . Revitalization of the preclinical and early detection program of breast cancer [in Spanish]. Communicational brief of the Ministry of Health. Ministry of Health of Cuba http://bvs.sld.cu/revistas/gme/pub/vol.7.(3)_08/p8.html. Accessed October 18, 2018.

[10] Guidelines for early detection of periodic inspection for breast cancer and cervical cancer in primary health care centers in Iraq [in Arabic]. Primary healthcare project. The Ministry of Health of Iraq and the USAID; 2013, 77pp.

[11] National guidelines for cancer management Kenya. The Ministry of Health of Republic of Kenya; 2013 http://kehpca.org/wp-content/uploads/National-Cancer-Treatment-Guidelines2.pdf. Accessed December 05, 2018.

[12] Guide to the early detection of breast and cervical cancers [in French]. The Ministry of Health of Morocco; 2011, 81pp.

[13] Early detection and management of breast symptoms. National Guideline for Primary Care Doctors and Family Physicians. Unit of Primary Prevention & Early Detection of Cancers. National Cancer Control Programme. Ministry of Health of Sri Lanka; 2014 http://www.nccp.health.gov.lk/images/PDF_FILES/GuideLine.pdf. Accessed December 5, 2018.

[14] Plan for the fight against cancer in Tunisia 2015‐2019 [in French]. Tunisian Republic Ministry of Health of Tunisia; 2015, 31pp.

[15] Ministry of Health and Family Welfare Bangladesh. National cancer control strategy and plan of action 2009–2015. Directorate General of Health Services. Dhaka: Non Communicable Diseases and Other Public Health Interventions; 2008.

[16] Bertram MY , Lauer JA , De Joncheere K , et al. Cost‐effectiveness thresholds: pros and cons. Bull World Health Organ. 2016;94:925‐930. 27994285 10.2471/BLT.15.164418 PMC5153921

[17] Hutubessy R , Chisholm D , Edejer TT‐T Generalized cost‐effectiveness analysis for national‐level priority‐setting in the health sector. Cost effectiveness and resource allocation. Cost Eff Resour Alloc. 2003;1:8. 14687420 10.1186/1478-7547-1-8 PMC320499

[18] Mandrik O , Ekwunife OI , Zielonke N , et al. What determines the effects and costs of breast cancer screening? A protocol of a systematic review of reviews. Syst Rev. 2017;6:122. 28659183 10.1186/s13643-017-0510-y PMC5490169

[19] Shea BJ , Hamel C , Wells GA , et al. AMSTAR is a reliable and valid measurement tool to assess the methodological quality of systematic reviews. J Clin Epidemiol. 2009;62:1013‐1020. 19230606 10.1016/j.jclinepi.2008.10.009

[20] Bell CM , Urbach DR , Ray JG , et al. Bias in published cost effectiveness studies: systematic review. BMJ. 2006;332:699‐703. 16495332 10.1136/bmj.38737.607558.80 PMC1410902

[21] GDP per capita (current US$). World Bank national accounts data, and OECD National Accounts data files. The World Bank https://data.worldbank.org/indicator/ny.gdp.pcap.cd. Accessed December 13, 2018.

[22] Barbieri M , Richardson G , Paisley S . The cost‐effectiveness of follow‐up strategies after cancer treatment: a systematic literature review. Br Med Bull. 2018;126:85‐100. 29659715 10.1093/bmb/ldy011

[23] Dinnes J , Moss S , Melia J , Blanks R , Song F , Kleijnen J . Effectiveness and cost‐effectiveness of double reading of mammograms in breast cancer screening: findings of a systematic review. Breast. 2001;10:455‐463. 14965624 10.1054/brst.2001.0350

[24] Kamal KM , Covvey JR , Dashputre A , et al. A systematic review of the effect of cancer treatment on work productivity of patients and caregivers. J Manag Care Spec Pharm. 2017;23:136‐162. 28125370 10.18553/jmcp.2017.23.2.136 PMC10397748

[25] Wagner TH . The effectiveness of mailed patient reminders on mammography screening: a meta‐analysis. Am J Prev Med. 1998;14:64‐70. 9476837 10.1016/s0749-3797(97)00003-2

[26] Baxter N . Preventive health care, 2001 update: should women be routinely taught breast self‐examination to screen for breast cancer? CMAJ. 2001;164:1837‐1846. 11450279 PMC81191

[27] Mandelblatt J , Saha S , Teutsch S , et al. The cost‐effectiveness of screening mammography beyond age 65 years: a systematic review for the U.S. Preventive Services Task Force. Ann Intern Med. 2003;139:835‐842. 14623621 10.7326/0003-4819-139-10-200311180-00011

[28] Ho C , Hailey D , Warburton R , MacGregor J , Pisano E , Joyce J . Digital mammography versus film‐screen mammography: technical, clinical and economic assessments. Technology report no 30. Canadian Coordinating Office for Health Technology Assessment; 2002.

[29] Health Quality Ontario . Ultrasound as an adjunct to mammography for breast cancer screening: a health technology assessment. Ont Health Technol Assess Ser. 2016;16:1‐71. PMC4947971 27468326

[30] Browall M , Forsberg C , Wengström Y . Assessing patient outcomes and cost‐effectiveness of nurse‐led follow‐up for women with breast cancer—have relevant and sensitive evaluation measures been used? J Clin Nurs. 2017;26:1770‐1786. 27487478 10.1111/jocn.13496

[31] Rashidian A , Barfar E , Hosseini H , Nosratnejad S , Barooti E . Cost effectiveness of breast cancer screening using mammography; a systematic review. Iran J Public Health. 2013;42:347‐357. 23785673 PMC3684720

[32] Yoo KB , Kwon JA , Cho E , et al. Is mammography for breast cancer screening cost‐effective in both Western and Asian countries?: results of a systematic review. Asian Pac J Cancer Prev. 2013;14:4141‐4149. 23991967 10.7314/apjcp.2013.14.7.4141

[33] Baron RC , Melillo S , Rimer BK , et al. Intervention to increase recommendation and delivery of screening for breast, cervical, and colorectal cancers by healthcare providers. A systematic review of provider reminders. Am J Prev Med. 2010;38:110‐117. 20117566 10.1016/j.amepre.2009.09.031

[34] Zelle SG , Baltussen RM . Economic analyses of breast cancer control in low‐ and middle‐income countries: a systematic review. Syst Rev. 2013;2:20. 23566447 10.1186/2046-4053-2-20 PMC3651267

[35] Meregaglia M , Cairns J . Economic evaluations of follow‐up strategies for cancer survivors: a systematic review and quality appraisal of the literature. Expert Rev Pharmacoecon Outcomes Res. 2015;15:913‐929. 26449255 10.1586/14737167.2015.1087316

[36] de Boer AG , Taskila T , Ojajarvi A , van Dijk FJ , Verbeek JH . Cancer survivors and unemployment: a meta‐analysis and meta‐regression. JAMA. 2009;301:753‐762. 19224752 10.1001/jama.2009.187

[37] Muka T , Imo D , Jaspers L , et al. The global impact of non‐communicable diseases on healthcare spending and national income: a systematic review. Eur J Epidemiol. 2015;30:251‐277. 25595318 10.1007/s10654-014-9984-2

[38] Chaker L , Falla A , van der Lee SJ , et al. The global impact of non‐communicable diseases on macro‐economic productivity: a systematic review. Eur J Epidemiol. 2015;30:357‐395. 25837965 10.1007/s10654-015-0026-5 PMC4457808

[39] Foster TS , Miller JD , Boye ME , Blieden MB , Gidwani R , Russell MW . The economic burden of metastatic breast cancer: a systematic review of literature from developed countries. Cancer Treat Rev. 2011;37:405‐415. 21477928 10.1016/j.ctrv.2010.12.008

[40] Jaspers L , Colpani V , Chaker L , et al. The global impact of non‐communicable diseases on households and impoverishment: a systematic review. Eur J Epidemiol. 2014;30:163‐188. 25527371 10.1007/s10654-014-9983-3

[41] Campbell JD , Ramsey SD . The costs of treating breast cancer in the US: a synthesis of published evidence. Pharmacoeconomics. 2009;27:199‐209. 19354340 10.2165/00019053-200927030-00003

[42] Arnold M . Simulation modeling for stratified breast cancer screening—a systematic review of cost and quality of life assumptions. BMC Health Serv Res. 2017;17:802. 29197417 10.1186/s12913-017-2766-2 PMC5712150

[43] van Hezewijk M , Elske van den Akker M , van de Velde C , Scholten AN , Hille E . Costs of different follow‐up strategies in early breast cancer: a review of the literature. Breast. 2012;21:693‐700. 23084960 10.1016/j.breast.2012.09.009

[44] Collins RF , Bekker HL , Dodwell DJ . Follow‐up care of patients treated for breast cancer: a structured review. Cancer Treat Rev. 2004;30:19‐35. 14766124 10.1016/S0305-7372(03)00141-5

[45] Lewis R , Neal RD , Williams NH , et al. Nurse‐led vs conventional physician‐led follow‐up for patients with cancer: systematic review. J Adv Nurs. 2009;65:706‐723. 19278415 10.1111/j.1365-2648.2008.04927.x

[46] Sun Y , Shigaki CL , Armer JM . Return to work among breast cancer survivors: A literature review. Support Care Cancer. 2017;25:709‐718. 27873016 10.1007/s00520-016-3446-1

[47] Posso M , Puig T , Carles M , Rue M , Canelo‐Aybar C , Bonfill X . Effectiveness and cost‐effectiveness of double reading in digital mammography screening: A systematic review and meta‐analysis. Eur J Radiol. 2017;96:40‐49. 29103474 10.1016/j.ejrad.2017.09.013

[48] Koleva‐Kolarova RG , Zhan Z , Greuter MJ , Feenstra TL , De Bock GH . Simulation models in population breast cancer screening: a systematic review. Breast. 2015;24:354‐363. 25906671 10.1016/j.breast.2015.03.013

[49] Baron RC , Rimer BK , Breslow RA , et al. Client‐directed interventions to increase community demand for breast, cervical, and colorectal cancer screening. a systematic review. Am J Prev Med. 2008;35:S34‐55. 18541187 10.1016/j.amepre.2008.04.002

[50] Li J , Shao Z . Mammography screening in less developed countries. Springerplus. 2015;4:615. 26543750 10.1186/s40064-015-1394-8 PMC4627993

[51] Abdel‐Aleem H , El‐Gibaly O , EL‐Gazzar A‐S , Al‐Attar G . Mobile clinics for women's and children's health. Cochrane Database Syst Rev. 2016 10.1002/14651858.CD009677.pub2. PMC9736774 27513824

[52] Evans DB , Tandon A , Murray CJ , Lauer JA . Comparative efficiency of national health systems: cross national econometric analysis. BMJ. 2001;323:307‐310. 11498486 10.1136/bmj.323.7308.307 PMC37316

[53] Bray F , Ferlay J , Soerjomataram I , Siegel RL , Torre LA , Jemal A . Global cancer statistics 2018: GLOBOCAN estimates of incidence and mortality worldwide for 36 cancers in 185 countries. CA Cancer J Clin. 2018. 10.3322/caac.21492 30207593

[54] Farmer P , Frenk J , Knaul FM , et al. Expansion of cancer care and control in countries of low and middle income: a call to action. Lancet. 2010;376:1186‐1193. 20709386 10.1016/S0140-6736(10)61152-X

[55] Anderson BO , Braun S , Lim S , Smith RA , Taplin S , Thomas DB . Early detection of breast cancer in countries with limited resources. Breast J. 2003;9(Suppl 2):S51‐S59. 12713497 10.1046/j.1524-4741.9.s2.4.x

[56] Drummond M , Barbieri M , Cook J , et al. Transferability of economic evaluations across jurisdictions: ISPOR Good Research Practices Task Force report. Value Health. 2009;12(4):409‐418. 19900249 10.1111/j.1524-4733.2008.00489.x

