# Supplementary material for: Systematic reviews as a “lens of evidence”: Determinants of cost‐effectiveness of breast cancer screening
Source: Cancer Med. 2019 Sep 30;8(18):7846–58. doi: 10.1002/cam4.2498 (PMC6912065; doi:10.1002/cam4.2498)
Supplement: Supplementary file 1 [file CAM4-8-7846-s001.docx]

## Appendix 1 Search strategy

**Search line in Pubmed via Medline (1 OR 2 OR 3) (search from commencement till August 2018):**

1. (breast AND (cancer OR cancers)) AND ((cost[Title/Abstract] OR costs[Title/Abstract] OR costing[Title/Abstract] OR cost -*[Title/Abstract] OR economic[Title/Abstract] OR economics[Title/Abstract] OR economical hospitalization[Title/Abstract] OR hospitalizations[Title/Abstract] OR hospitalisation[Title/Abstract] OR absenteeism[Title/Abstract] OR productivity[Title/Abstract] OR productivities[Title/Abstract])) Filters: Review
2. (Breast Neoplasms[MeSH Terms]) AND ((Health Resources[MeSH Terms]) OR (Costs and Cost Analysis[MeSH Terms])) OR Health Resources/economics[MeSH Terms]) OR Health Resources/utilization[MeSH Terms] Filters: Review

**Additional focused search including the grey literature:**

Journals: Plos One, Cancer, Cancer Epidemiology, the Breast, Breast Cancer, European Journal of Surgical Oncology, the Lancet Oncol, JAMA Oncology, JAMA.

Data bases: Google Scholar (“breast cancer cost”), first 300 hits

Reports and other grey literature: Blue Cross and Blue Shield Association; The National Institute for Health and Care Excellence (NICE), The Canadian Agency for Drugs and Technologies in Health (CADTH, Canada), Institute for Quality and Efficiency in Health Care (IQWIG, Germany), NHS Quality Improvement Scotland; Canadian Coordinating Office for Health Technology Assessment (CCOHTA); Comite d’Evaluation et de Diffusion des Innovations Technologiques (CEDIT); Agence Nationale d'Accréditation et d'Evaluation en Santé (ANAES); the American Society of Clinical Oncology (ASCO), the American Cancer Society, the International Agency for Research on Cancer, the European Society for Medical Oncology, International Society for Pharmacoeconomics and Outcomes Research (ISPOR).
