# Supplementary material for: Systematic reviews as a “lens of evidence”: Determinants of cost‐effectiveness of breast cancer screening
Source: Cancer Med. 2019 Sep 30;8(18):7846–58. doi: 10.1002/cam4.2498 (PMC6912065; doi:10.1002/cam4.2498)
Supplement: Supplementary file 2 [file CAM4-8-7846-s002.docx]

# Appendix 2. Approach to transferability assessment

The approach to transferability assessment was developed based on the available literature on transferability^1-4^. Since the available instrument served for assessment of transferability of health economic outcomes were designed for original studies but not the reviews, we developed the decision framework presented on the Figure A1.

The transferability frameworks available for assessment of original studies define Quality as the first exclusion criterion^1^, which was also included into our framework. Since reviews report the secondary evidence, the transparency of reporting in both the reviews and studies is necessary to have transferable results, what is captured by the component “Uncertainty”. Variability component captures the differences in the outcomes and values reported in the reviews. While costs are considered not transferable^2^, some determinants of costs (for example, productivity losses) can be potentially transferable between the jurisdictions of interests. We considered that the conclusions of the reviews were transferable to comparable jurisdictions, if the reviews reported low variability and uncertainty of the results, and considered the quality of original evidence as high or sufficient. If all three components in our decision scheme were judged as poor, the transferability was concluded to be “very low”.

If exact definitions were not applied in the reviews, we referred to the following agreed upon definitions: (a) “low variability” - not significant differences in outcomes and their values observed among the included studies able to retrieve a conclusion as either a summarized value or a trend;

(b) “high uncertainty” - a lack of confidence in the generalized conclusions because of unavailable or limited evidence or its low quality.

F**igure A1. Schematic presentation of transferability assessment**

No

Yes

Low transferability

No

No

No

Yes

Quality

Not assessed/ not reported

Low or very low

To the comparable jurisdictions (eg if only or mostly HIC included, - to HIC)

Low transferability

Low transferability

Not clear/ not reported

High due to any factors /significant across the studies

Very small number of studies

Uncertainty

Low transferability

Not clear/ not reported

High due to any factors /significant across the studies

Variability

Yes

Yes

**References**

1. Welte R, Feenstra T, Jager H, Leidl R. A decision chart for assessing and improving the transferability of economic evaluation results between countries. *PharmacoEconomics* 2004;**22**: 857-76.

2. Goeree R, Burke N, O'Reilly D, Manca A, Blackhouse G, Tarride JE. Transferability of economic evaluations: approaches and factors to consider when using results from one geographic area for another. *Current medical research and opinion* 2007;**23**: 671-82.

3. Goeree R, He J, O'Reilly D, Tarride JE, Xie F, Lim M, Burke N. Transferability of health technology assessments and economic evaluations: a systematic review of approaches for assessment and application. *ClinicoEconomics and outcomes research : CEOR* 2011;**3**: 89-104.

4. Mandrik O, Knies S, Kalo Z, Severens JL. REVIEWING TRANSFERABILITY IN ECONOMIC EVALUATIONS ORIGINATING FROM EASTERN EUROPE. *International journal of technology assessment in health care* 2015;**31**: 434-41.
