# Supplementary material for: Systematic reviews as a “lens of evidence”: Determinants of cost‐effectiveness of breast cancer screening
Source: Cancer Med. 2019 Sep 30;8(18):7846–58. doi: 10.1002/cam4.2498 (PMC6912065; doi:10.1002/cam4.2498)
Supplement: Supplementary file 3 [file CAM4-8-7846-s003.docx]

# Appendix 3. Articles excluded by each exclusion criterion

## Not on breast cancer

1. Shen C, Chien CR, Geynisman DM, Smieliauskas F, Shih YCT. A review of economic impact of targeted oral anticancer medications. Expert Review of Pharmacoeconomics and Outcomes Research 2014;14: 45-69.

2. Velentzis LS, Salagame U, Canfell K. Menopausal hormone therapy: a systematic review of cost-effectiveness evaluations. BMC health services research 2017;17: 326.

3. Soejima T, Kamibeppu K. Are cancer survivors well-performing workers? A systematic review. Asia-Pacific Journal of Clinical Oncology 2016;12: e383-e97.

## Only one country

1. Pisu M, Azuero A, McNees P, Burkhardt J, Benz R, Meneses K. The out of pocket cost of breast cancer survivors: a review. Journal of cancer survivorship : research and practice 2010;4: 202-9.

2. Parisi M, Pelletier C, Cherepanov D, Broder MS. Outcomes research examining treatments, quality of life and costs in HER2-negative and triple-negative metastatic breast cancer: A systematic literature review. Journal of Comparative Effectiveness Research 2018;7: 67-83.

## Not a systematic search of original evidence

1. Albert US, Altland H, Duda V, Engel J, Geraedts M, Heywang-Kobrunner S, Holzel D, Kalbheim E, Koller M, Konig K, Kreienberg R, Kuhn T, et al. [Guideline for the Early Detection of Breast Cancer in Germany 2008. Recommendations from the short version]. *Der Chirurg; Zeitschrift fur alle Gebiete der operativen Medizen* 2008;**79**: 589-94.

2. Albert US, Altland H, Duda VF, Engel J, Geraedts M, Heywang-Köbrunner S, Hölzel D, Kalbheim E, Koller M, König K, Kreienberg R, Kühn T, et al. Early detection of breast cancer in Germany. Guideline 2008. *Onkologe* 2008;**14**: 461-77.

3. Alvarez Hernandez C, Vich Perez P, Brusint B, Cuadrado Rouco C, Diaz Garcia N, Robles Diaz L. [Update of breast cancer in Primary Care (III/V)]. *Semergen* 2014;**40**: 460-72.

4. Alvarez-Hernandez C, Brusint B, Vich P, Diaz-Garcia N, Cuadrado-Rouco C, Hernandez-Garcia M. [Update of breast cancer in primary care (IV/V)]. *Semergen* 2015;**41**: 34-47.

1.

5. Bahadursingh S, Maharaj R, Harnarayan P, Cawich SO, Yearwood M, Naraynsingh V. Mammographic screening: Is it relevant to developing countries? *Current Medicine Research and Practice* 2014;**4**: 168-70.

6. Baker S, Wall M, Bloomfield A. Breast cancer screening for women aged 40 to 49 years--what does the evidence mean for New Zealand? *The New Zealand medical journal* 2005;**118**: U1628.

7. Barth J. Outpatient chemotherapy - Discover the possibilities. *Krankenhauspharmazie* 2004;**25**: 94-101.

8. Bellon A, Perez-Garcia G, Coverdale JH, Chacko RC. Seizures associated with levofloxacin: Case presentation and literature review. *European Journal of Clinical Pharmacology* 2009;**65**: 959-62.

9. Brown DW, French MT, Schweitzer ME, McGeary KA, McCoy CB, Ullman SG. Economic evaluation of breast cancer screening: A review. *Cancer practice* 1999;**7**: 28-33.

10. Brown ML, Fintor L. Cost-effectiveness of breast cancer screening: preliminary results of a systematic review of the literature. *Breast cancer research and treatment* 1993;**25**: 113-8.

11. Bryson HM, Plosker GL. Tamoxifen: a review of pharmacoeconomic and quality-of-life considerations for its use as adjuvant therapy in women with breast cancer. *PharmacoEconomics* 1993;**4**: 40-66.

12. Bottomley A, Therasse P. Quality of life in patients undergoing systemic therapy for advanced breast cancer. *Lancet Oncology* 2002;**3**: 620-8.

13. Conde DM, Pinto-Neto AM, De Freitas Jr R, Aldrighi JM. Quality of life in breast cancer survivors. *Revista Brasileira de Ginecologia e Obstetricia* 2006;**28**: 195-204.

14. Corry JF, Lonning PE. Systemic therapy in breast cancer: efficacy and cost utility. *PharmacoEconomics* 1994;**5**: 198-212.

15. de Haes JC, de Koning HJ, van Oortmarssen GJ, van Agt HM, de Bruyn AE, van Der Maas PJ. The impact of a breast cancer screening programme on quality-adjusted life-years. *Int J Cancer* 1991;**49**: 538-44.

16. Delaney G, Barton M, Jacob S. Estimation of an optimal radiotherapy utilization rate for breast carcinoma: a review of the evidence. *Cancer* 2003;**98**: 1977-86.

17. Diaby V, Tawk R, Sanogo V, Xiao H, Montero AJ. A review of systematic reviews of the cost-effectiveness of hormone therapy, chemotherapy, and targeted therapy for breast cancer. *Breast cancer research and treatment* 2015;**151**: 27-40.

18. Elixhauser A. Costs of breast cancer and the cost-effectiveness of breast cancer screening. *International journal of technology assessment in health care* 1991;**7**: 604-15.

19. Fraser NM, Clarke PR. Cost-effectiveness of breast cancer screening. *The Breast* 1992;**1**: 169-72.

20. Frederix GWJ, Severens JL, Hövels AM, Raaijmakers JAM, Schellens JHM. The cloudy crystal ball of cost-effectiveness studies. *Value in Health* 2013;**16**: 1100-2.

21. Fregene A, Newman LA. Breast cancer in sub-Saharan Africa: how does it relate to breast cancer in African-American women? *Cancer* 2005;**103**: 1540-50.

22. Full Field Digital Mammography versus Computed Radiography for Breast Cancer Screening: A Clinical and Cost-Effectiveness Review. CADTH, 2008.

23. Gelband H, Sankaranarayanan R, Gauvreau CL, Horton S, Anderson BO, Bray F, Cleary J, Dare AJ, Denny L, Gospodarowicz MK, Gupta S, Howard SC, et al. Costs, affordability, and feasibility of an essential package of cancer control interventions in low-income and middle-income countries: Key messages from Disease Control Priorities, 3rd edition. *The Lancet* 2016;**387**: 2133-44.

24. Gonzalez San Segundo C, Calvo Manuel FA, Santos Miranda JA. [Delays and treatment interruptions: difficulties in administering radiotherapy in an ideal time-period]. *Clinical & translational oncology : official publication of the Federation of Spanish Oncology Societies and of the National Cancer Institute of Mexico* 2005;**7**: 47-54.

25. Goodwin PJ. Economics, quality of life and breast cancer outcomes - Is a balance possible? *Breast (Edinburgh, Scotland)* 2001;**10**: 190-8.

26. Hanly P, Soerjomataram I, Sharp L. Measuring the societal burden of cancer: The cost of lost productivity due to premature cancer-related mortality in Europe. *International Journal of Cancer* 2016;**136**: E136-E45.

27. Hao Y, Wolfram V, Cook J. A structured review of health utility measures and elicitation in advanced/metastatic breast cancer. *ClinicoEconomics and Outcomes Research* 2016;**8**: 293-303.

28. Hedden L, O'Reilly S, Lohrisch C, Chia S, Speers C, Kovacic L, Taylor S, Peacock S. Assessing the real-world cost-effectiveness of adjuvant trastuzumab in HER-2/neu positive breast cancer. *Oncologist* 2012;**17**: 164-71.

29. Horton DA. Breast cancer screening of women age 65 or older - A review of the evidence on specificity, effectiveness and compliance. *Breast (Edinburgh, Scotland)* 1993;**2**: 64-6.

30. Jeyakumar A, Younis T. Trastuzumab for HER2-positive metastatic breast cancer: Clinical and economic considerations. *Clinical Medicine Insights: Oncology* 2012;**6**: 179-87.

31. Jones L, Hawkins N, Westwood M, Wright K, Richardson G, Riemsma R. Systematic review of the clinical effectiveness and cost-effectiveness of capecitabine (Xeloda) for locally advanced and/or metastatic breast cancer. *Health Technol Assess* 2004;**8**: iii, xiii-xvi, 1-143.

32. Kattlove H, Liberati A, Keeler E, Brook RH. Benefits and costs of screening and treatment for early breast cancer: Development of a basic benefit package. *Journal of the American Medical Association* 1995;**273**: 142-8.

33. Kilian R, Porzsolt F. When to recommend and to pay for first-line adjuvant breast cancer treatment? A structured review of the literature. *Breast (Edinburgh, Scotland)* 2005;**14**: 636-42.

34. Lamb HM, Wiseman LR. Docetaxel. A pharmacoeconomic review of its use in the treatment of metastatic breast cancer. *PharmacoEconomics* 1998;**14**: 447-59.

35. Lavina H, Lickley A. Primary breast cancer in the elderly. *Canadian Journal of Surgery* 1997;**40**: 341-51.

36. Lebovic GS, Hollingsworth A, Feig SA. Risk assessment, screening and prevention of breast cancer: A look at cost-effectiveness. *Breast (Edinburgh, Scotland)* 2010;**19**: 260-7.

37. Lickley HL. Primary breast cancer in the elderly. *Canadian journal of surgery Journal canadien de chirurgie* 1997;**40**: 341-51.

38. Lo-Fo-Wong DN, Sitnikova K, Sprangers MA, de Haes HC. Predictors of Health Care Use of Women with Breast Cancer: A Systematic Review. *Breast J* 2015;**21**: 508-13.

39. Lüftner D, Lux MP, Maass N, Schütz F, Schwidde I, Fasching PA, Fehm T, Janni W, Kümmel S, Kolberg HC. Advances in breast cancer - Looking back over the year. *Geburtshilfe und Frauenheilkunde* 2012;**72**: 1117-29.

40. Lux MP, Hildebrandt T, Bani M, Loehberg CR, Schrauder MG, Rauh C, Jud SM, Fasching PA, Hartmann A, Beckmann MW. Health economic evaluation of different decision aids for the individualised treatment of patients with breast cancer. *Geburtshilfe und Frauenheilkunde* 2013;**73**: 599-610.

41. Mackey JR, Joy AA. Skeletal health in postmenopausal survivors of early breast cancer. Int J Cancer 2005;114: 1010-5.

42. McKoy JM, Fitzner KA, Edwards BJ, Alkhatib M, Tigue CC, Nonzee NJ, Bolden CR, Bennett CL. Cost considerations in the management of cancer in the older patient. *Oncology (Williston Park)* 2007;**21**: 851-7; discussion 8, 60, 62 passim.

43. Pallis A, Tsiantou V, Simou E, Maniadakis N. Pharmacoeconomic considerations in the treatment of breast cancer. *ClinicoEconomics and Outcomes Research* 2010;**2**: 47-61.

44. Portable and Mobile Mammography Screening Services. CADTH, 2007.

45. Robertson C, Arcot Ragupathy SK, Boachie C, Dixon JM, Fraser C, Hernández R, Heys S, Jack W, Kerr G, Lawrence G, MacLennan G, Maxwell A, et al. The clinical effectiveness and costeffectiveness of different surveillance mammography regimens after the treatment for primary breast cancer: Systematic reviews, registry database analyses and economic evaluation. *Health Technology Assessment* 2011;**15**.

46. Sabatino SA, Habarta N, Baron RC, Coates RJ, Rimer BK, Kerner J, Coughlin SS, Kalra GP, Chattopadhyay S. Interventions to Increase Recommendation and Delivery of Screening for Breast, Cervical, and Colorectal Cancers by Healthcare Providers. Systematic Reviews of Provider Assessment and Feedback and Provider Incentives. *American Journal of Preventive Medicine* 2008;**35**.

47. Sankaranarayanan R. Screening for cancer in low- and middle-income countries. *Annals of Global Health* 2014;**80**: 412-7.

48. Smith TJ, Davidson NE, Schapira DV, Grunfeld E, Muss HB, Vogel Iii VG, Somerfield MR. American Society of Clinical Oncology 1998 update of recommended breast cancer surveillance guidelines. *Journal of Clinical Oncology* 1999;**17**: 1080-2.

49. Takeda AL, Jones J, Loveman E, Tan SC, Clegg AJ. The clinical effectiveness and cost-effectiveness of gemcitabine for metastatic breast cancer: a systematic review and economic evaluation. *Health Technol Assess* 2007;**11**: iii, ix-xi, 1-62.

50. Trudeau M, Charbonneau F, Gelmon K, Laing K, Latreille J, Mackey J, McLeod D, Pritchard K, Provencher L, Verma S. Selection of adjuvant chemotherapy for treatment of node-positive breast cancer. *The Lancet Oncology* 2005;**6**: 886-98.

## No individual quality assessment for each included study

1. Barratt AL, Irwig LM, Glasziou PP, Salkeld GP, Houssami N. Benefits, harms and costs of screening mammography in women 70 years and over: A systematic review. Medical Journal of Australia 2002;176: 266-71.

2. Gandhi S, Verma S, Ethier JL, Simmons C, Burnett H, Alibhai SM. A systematic review and quality appraisal of international guidelines for early breast cancer systemic therapy: Are recommendations sensitive to different global resources? Breast (Edinburgh, Scotland) 2015;24: 309-17.

3. Landercasper J, Tafra L. The relationship between quality and cost during the perioperative breast cancer episode of care. Breast (Edinburgh, Scotland) 2010;19: 289-96.

4. Norum J. The cost-effectiveness issue of adjuvant trastuzumab in early breast cancer. Expert opinion on pharmacotherapy 2006;7: 1617-25.

5. Muszbek N, Koncz T, P VH, Adany R. [Economic evaluation of population-based mass screening for the early detection of cancer: a systematic review]. Magy Onkol 2002;46: 119-29.

## Does not report target outcomes

1. Baron RC, Rimer BK, Breslow RA, Coates RJ, Kerner J, Melillo S, Habarta N, Kalra GP, Chattopadhyay S, Wilson KM, Lee NC, Mullen PD, et al. Client-Directed Interventions to Increase Community Demand for Breast, Cervical, and Colorectal Cancer Screening. A Systematic Review. *American Journal of Preventive Medicine* 2008;**35**.

2. Barbieri M, Weatherly HL, Ara R, Basarir H, Sculpher M, Adams R, Ahmed H, Coles C, Guerrero-Urbano T, Nutting C, Powell M. What is the quality of economic evaluations of non-drug therapies? A systematic review and critical appraisal of economic evaluations of radiotherapy for cancer. *Applied health economics and health policy* 2014;**12**: 497-510.

3. Baxi SS, Kale M, Keyhani S, Roman BR, Yang A, Derosa AP, et al. Overuse of Health Care Services in the Management of Cancer: A Systematic Review. Medical Care. 2017;55(7):723-33.

4. Blank PR, Dedes KJ, Szucs TD. Cost effectiveness of cytotoxic and targeted therapy for metastatic breast cancer: A critical and systematic review. *PharmacoEconomics* 2010;**28**: 629-47.

5. Boswell KA, Wang X, Shah MV, Aapro MS. Disease burden and treatment outcomes in second-line therapy of patients with estrogen receptor-positive (ER+) advanced breast cancer: A review of the literature. *Breast (Edinburgh, Scotland)* 2012;**21**: 701-6.

6. Brown J, Sculpher M. Benefit valuation in economic evaluation of cancer therapies: A systematic review of the published literature. *PharmacoEconomics* 1999;**16**: 17-31.

7. Chan AL, Leung HW, Lu CL, Lin SJ. Cost-effectiveness of trastuzumab as adjuvant therapy for early breast cancer: a systematic review. *The Annals of pharmacotherapy* 2009;**43**: 296-303.

8. Cheng KKF, Lim YTE, Koh ZM, Tam WWS. Home-based multidimensional survivorship programmes for breast cancer survivors. *The Cochrane database of systematic reviews* 2017;**8**: Cd011152.

9. Dvortsin E, Gout-Zwart J, Eijssen ELM, Van Brussel J, Postma MJ. Comparative cost-effectiveness of drugs in early versus late stages of cancer; Review of the literature and a case study in breast cancer. *PloS one* 2016;**11**.

10. [P14-03] Einladungsschreiben und Entscheidungshilfe zum Mammographie-Screening. IQWiG, 2014.

11. Ellis MJ, Rigden CE. Initial versus sequential adjuvant aromatase inhibitor therapy: a review of the current data. *Curr Med Res Opin* 2006;**22**: 2479-87.

12. Farquhar C, Marjoribanks J, Basser R, Hetrick S, Lethaby A. High dose chemotherapy and autologous bone marrow or stem cell transplantation versus conventional chemotherapy for women with metastatic breast cancer. *The Cochrane database of systematic reviews* 2005: Cd003142.

13. Ferrusi IL, Leighl NB, Kulin NA, Marshall DA. Do economic evaluations of targeted therapy provide support for decision makers? *The American journal of managed care* 2011;**17 Suppl 5 Developing**.

14. Ferrusi IL, Marshall DA, Kulin NA, Leighl NB, Phillips KA. Looking back at 10 years of trastuzumab therapy: What is the role of HER2 testing? A systematic review of health economic analyses. *Personalized Medicine* 2009;**6**: 193-215.

15. Fleeman N, Martin Saborido C, Payne K, Boland A, Dickson R, Dundar Y, Fernandez Santander A, Howell S, Newman W, Oyee J, Walley T. The clinical effectiveness and cost-effectiveness of genotyping for CYP2D6 for the management of women with breast cancer treated with tamoxifen: a systematic review. *Health Technol Assess* 2011;**15**: 1-102.

16. Frederix GW, Severens JL, Hovels AM, Raaijmakers JA, Schellens JH. Reviewing the cost-effectiveness of endocrine early breast cancer therapies: influence of differences in modeling methods on outcomes. *Value in health : the journal of the International Society for Pharmacoeconomics and Outcomes Research* 2012;**15**: 94-105.

17. Gagliardi A, Grunfeld E, Evans WK. Evaluation of diagnostic assessment units in oncology: a systematic review. *Journal of clinical oncology : official journal of the American Society of Clinical Oncology* 2004;**22**: 1126-35.

18. Ghislain I, Zikos E, Coens C, Quinten C, Balta V, Tryfonidis K, Piccart M, Zardavas D, Nagele E, Bjelic-Radisic V, Cardoso F, Sprangers MAG, et al. Health-related quality of life in locally advanced and metastatic breast cancer: methodological and clinical issues in randomised controlled trials. *The Lancet Oncology* 2016;**17**: e294-e304.

19. Greenberg D, Earle C, Fang CH, Eldar-Lissai A, Neumann PJ. When is cancer care cost-effective? a systematic overview of cost-utility analyses in oncology. *Journal of the National Cancer Institute* 2010;**102**: 82-8.

20. Gooiker GA, van Gijn W, Post PN, van de Velde CJ, Tollenaar RA, Wouters MW. A systematic review and meta-analysis of the volume-outcome relationship in the surgical treatment of breast cancer. Are breast cancer patients better of with a high volume provider? *European journal of surgical oncology : the journal of the European Society of Surgical Oncology and the British Association of Surgical Oncology* 2010;**36 Suppl 1**: S27-35.

21. Hind D, Ward S, De Nigris E, Simpson E, Carroll C, Wyld L. Hormonal therapies for early breast cancer: systematic review and economic evaluation. *Health Technol Assess* 2007;**11**: iii-iv, ix-xi, 1-134.

22. John-Baptiste AA, Wu W, Rochon P, Anderson GM, Bell CM. A systematic review and methodological evaluation of published cost-effectiveness analyses of aromatase inhibitors versus tamoxifen in early stage breast cancer. *PloS one* 2013;**8**: e62614.

23. Karlsson G, Nygren P, Glimelius B. Economic aspects of chemotherapy. *Acta Oncologica* 2001;**40**: 412-33.

24. Karnon J. Cost-effectiveness of letrozole, anastrozole and exemestane for early adjuvant breast cancer. *Expert Review of Pharmacoeconomics and Outcomes Research* 2007;**7**: 143-53.

25. Karnon J. Aromatase inhibitors in breast cancer: a review of cost considerations and cost effectiveness. *PharmacoEconomics* 2006;**24**: 215-32.

26. Lafranconi A, Pylkkänen L, Deandrea S, Bramesfeld A, Lerda D, Neamtiu L, Saz-Parkinson Z, Posso M, Rigau D, Alonso-Coello P, Martinez-Zapata MJ. Intensive follow-up for women with breast cancer: Review of clinical, economic and patient's preference domains through evidence to decision framework. *Health and Quality of Life Outcomes* 2017;**15**.

27. Lewis R, Bagnall AM, King S, Woolacott N, Forbes C, Shirran L, Duffy S, Kleijnen J, ter Riet G, Riemsma R. The clinical effectiveness and cost-effectiveness of vinorelbine for breast cancer: a systematic review and economic evaluation. *Health Technol Assess* 2002;**6**: 1-269.

28. Lodge M, Corbex M. Establishing an evidence-base for breast cancer control in developing countries. *Breast (Edinburgh, Scotland)* 2011;**20 Suppl 2**: S65-9.

29. Lister-Sharp D, McDonagh MS, Khan KS, Kleijnen J. A rapid and systematic review of the effectiveness and cost-effectiveness of the taxanes used in the treatment of advanced breast and ovarian cancer. *Health Technol Assess* 2000;**4**: 1-113.

30. Ly M, Antoine M, Andre F, Callard P, Bernaudin JF, Diallo DA. [Breast cancer in Sub-Saharan African women: review]. *Bull Cancer* 2011;**98**: 797-806.

31. Mansfield C, Tangka FKL, Ekwueme DU, Smith JL, Guy GP, Li C, Brett Hauber A. Stated preference for cancer screening: A systematic review of the literature, 1990-2013. *Preventing Chronic Disease* 2016;**13**.

32. Miao H, Hartman M, Bhoo-Pathy N, Lee SC, Taib NA, Tan EY, Chan P, Moons KG, Wong HS, Goh J, Rahim SM, Yip CH, et al. Predicting survival of de novo metastatic breast cancer in Asian women: systematic review and validation study. *PloS one* 2014;**9**: e93755.

33. Monten C, Veldeman L, Verhaeghe N, Lievens Y. A systematic review of health economic evaluation in adjuvant breast radiotherapy: Quality counted by numbers. *Radiotherapy and oncology : journal of the European Society for Therapeutic Radiology and Oncology* 2017;**125**: 186-92.

34. Monten C, Lievens Y. Adjuvant breast radiotherapy: How to trade-off cost and effectiveness? *Radiotherapy and Oncology* 2017.

35. Najafipour F, Hamouzadeh P, Arabloo J, Mobinizadeh M, Norouzi A. Safety, effectiveness and economic evaluation of intra-operative radiation therapy: A systematic review. *Medical Journal of the Islamic Republic of Iran* 2015;**29**: 784-93.

36. Nerich V, Saing S, Gamper EM, Kemmler G, Daval F, Pivot X, Holzner B. Cost–utility analyses of drug therapies in breast cancer: a systematic review. *Breast cancer research and treatment* 2016;**159**: 407-24.

37. Parkinson B, Pearson SA, Viney R. Economic evaluations of trastuzumab in HER2-positive metastatic breast cancer: a systematic review and critique. *The European journal of health economics : HEPAC : health economics in prevention and care* 2014;**15**: 93-112.

38. Pickard AS, Wilke CT, Lin HW, Lloyd A. Health utilities using the EQ-5D in studies of cancer. *PharmacoEconomics* 2007;**25**: 365-84.

39. Pouwels XGLV, Ramaekers BLT, Joore MA. Reviewing the quality, health benefit and value for money of chemotherapy and targeted therapy for metastatic breast cancer. *Breast cancer research and treatment* 2017;**165**: 485-98.

41. Reiazi R, Norozi A, Etedadialiabadi M. A literature survey on cost-effectiveness of proton beam therapy in the management of breast cancer patients. *Iranian Journal of Cancer Prevention* 2015;**8**.

42. Schiller-Fruhwirth IC, Jahn B, Arvandi M, Siebert U. Cost-Effectiveness Models in Breast Cancer Screening in the General Population: A Systematic Review. *Appl Health Econ Health Policy* 2017;**15**: 333-51.

43. Schommer JC, Castellanos JW, Sanchez LD, Wagner S, Ye X. Opportunities for filling gaps in breast and lung cancer outcomes research. *Drug Information Journal* 2005;**39**: 335-44.

44. Smieliauskas F, Chien CR, Shen C, Geynisman DM, Shih YCT. Cost-effectiveness analyses of targeted oral anti-cancer drugs: A systematic review. *PharmacoEconomics* 2014;**32**: 651-80.

45. Tartari F, Conti A, Cerqueti R. Assessing the relationship between toxicity and economic cost of oncological target agents: A systematic review of clinical trials. *PloS one* 2017;**12**.

46. Verma V, Mishra MV, Mehta MP. A systematic review of the cost and cost-effectiveness studies of proton radiotherapy. *Cancer* 2016;**122**: 1483-501.

47. Ward S, Simpson E, Davis S, Hind D, Rees A, Wilkinson A. Taxanes for the adjuvant treatment of early breast cancer: systematic review and economic evaluation. *Health Technol Assess* 2007;**11**: 1-144.

48. Williams C, Brunskill S, Altman D, Briggs A, Campbell H, Clarke M, Glanville J, Gray A, Harris A, Johnston K, Lodge M. Cost-effectiveness of using prognostic information to select women with breast cancer for adjuvant systemic therapy. *Health Technol Assess* 2006;**10**: iii-iv, ix-xi, 1-204.

49. Winn AN, Ekwueme DU, Guy GP, Neumann PJ. Cost-Utility Analysis of Cancer Prevention, Treatment, and Control: A Systematic Review. *American Journal of Preventive Medicine* 2016;**50**: 241-8.

50. Zeng Y, Huang M, Cheng AS, Zhou Y, So WK. Meta-analysis of the effects of exercise intervention on quality of life in breast cancer survivors. *Breast cancer (Tokyo, Japan)* 2014;**21**: 262-74.

51. Zagouri F, Liakou P, Bartsch R, Peccatori FA, Tsigginou A, Dimitrakakis C, Zografos GC, Dimopoulos MA, Azim HA, Jr. Discrepancies between ESMO and NCCN breast cancer guidelines: An appraisal. *Breast (Edinburgh, Scotland)* 2015;**24**: 513-23.

## Duplicates with already included results

1. Lewis RA, Neal RD, Williams NH, France B, Hendry M, Russell D, Hughes DA, Russell I, Stuart NS, Weller D, Wilkinson C. Follow-up of cancer in primary care versus secondary care: systematic review. The British journal of general practice : the journal of the Royal College of General Practitioners 2009;59.

2. [P14-03] Einladungsschreiben und Entscheidungshilfe zum Mammographie-Screening. IQWiG, 2014.
