# Supplementary material for: Systematic reviews as a “lens of evidence”: Determinants of cost‐effectiveness of breast cancer screening
Source: Cancer Med. 2019 Sep 30;8(18):7846–58. doi: 10.1002/cam4.2498 (PMC6912065; doi:10.1002/cam4.2498)
Supplement: Supplementary file 4 [file CAM4-8-7846-s004.docx]

# Appendix 4. General and methodologic characteristics of the included systematic reviews

**Appendix 4a. Characteristics of the systematic reviews on breast cancer costs**

| **Author (first), year** | **(a) Department ^1^**  **(b) Funding** | **(a) Year^2^**  **(b) Search/ target setting**  **(c) Type^3^**  **(d) N^3^** | **Key Review questions** | **(a) Level**  **(b) Currency conversion** | **Quality assessment** | **Countries included** | **(a) Variability ^4^**  **(b) Uncertainty^4^**  **(c) Transferability^5^** | **AMSTAR** |
| --- | --- | --- | --- | --- | --- | --- | --- | --- |
| Collins, 2004^1^ | (a) Health Sciences  (b) Not stated | (a) 2001  (b) World  (c) Not clear  (d) 3 | Effectiveness of follow-up strategies | (a) Micro  (b) No | Not standardized | Not clear | (a) Not clear  (b) Not clear  (c) Low | 4 |
| Boer, 2009^2^ | (a) Public Health/ Medical  (b) State or non-for profit | (a) 2008  (b) World  (c) Empirical  (d) 10 | Unemployment  among adult cancer survivors  and the influence of prognostic  factors on unemployment | (a) Micro  (b) NA | Methodological Index for Non-randomized Studies | Canada, Finland, NL, Norway, USA | (a) Not for the risk of unemployment  (b) Low  (c) HIC | 5 |
| Campbell, 2009^3^ | (a) Medical  (b) Industry | (a) 2007  (b) USA  (c) Cost-of illness  (d) 29 | Cost of BC | (a) Micro  (b) NA | Not reported | USA | (a) Significant across the studies  (b) High due to heterogeneity  (c) Very low | 3 |
| Lewis, 2009^4^ | (a) Public Health  (b) State or non-for profit | (a) 2007  (b) World  (c) Trials  (d) 2 | Cost and CEA of nurse-led follow-up of patients with cancer | (a) Micro  (b) No | (1) Down and Black, (2) Drummond checklists | Sweden, UK | (a) Across studies in methods and presentation  (b) Not discussed, limited number of studies  (c) Low | 6 |
| Foster, 2011^5^ | (a) Consulting  (b) Industry | (a) 2010  (b) Developed countries  (c) Economic studies  (d) 34 | Economic impact of metastatic breast cancer and its treatment | (a) Macro and micro  (b) No | Not reported | Australia, Canada, France, Germany, Italy,  Spain, Sweden, Switzerland, the UK, and the US | (a) Not clear  (b) Due to possible conflict of interest  (c) Very low | 2 |
| van Hezewijk, 2012^6^ | (a) Medical  (b) Not stated | (a) 2011  (b) World  (c) RCT, non RCT, observational and modeling  (d) 11 | Costs of follow-up strategies | (a) Micro  (b) No | Not standardized (RCTs) | Finland, NL, Sweden, Spain, UK (RCTs); Australia, France, UK, the USA (non-RCTs) | (a) Across studies in interventions and methods  (b) Limited number of studies  (c) Low | 3 |
| Jaspers, 2014^7^ | (a) Epidemiology  (b) Mixed | (a) 2014  (b) World  (c) RCT, non RCT, observational, ecological, systematic reviews and modeling  (d) 21 | Impact of NCDs on households and poverty | (a) Micro  (b) USD using exchange rate or CPI, 2013 | Newcastle - Otawa Scale | Australia, Canada, China, Norway, NZ, Pakistan, Sweden, USA | (a) Significant across the studies  (b) High due to heterogeneity  (c) Low | 4 |
| Muka, 2015^8^ | (a) Epidemiology  (b) State or non-for profit | (a) 2014  (b) World  (c) Any  (d) 21 | Economic burden of NCDs | (a) Macro  (b) USD in 2013 using exchange rate | Newcastle-Otawa Scale | Belgium, France, Germany, Japan, Sweden, USA | (a) Significant across the studies  (b) Not discussed  (c) Low | 4 |
| Chaker, 2015^9^ | (a) Epidemiology  (b) Mixed | (a) 2011  (b) World  (c) RCT, non RCT, observational, ecological, systematic reviews and modeling  (d) 44 | Productivity impact of NCDs | (a) Macro  (b) USD in 2013 using exchange rate | Newcastle-Otawa Scale | Brazil, Belgium, Canada, Denmark, France, Finland, Germany, NL, Norway, NZ, Peru, South Korea, Spain, Sweden, USA | (a) Significant across the studies  (b) For LMICs  (c) Low | 4 |
| Meregaglia, 2015^10^ | (a) Public Health  (b) No | (a) 2014  (b) World  (c) Economic evaluations  (d) 10 | Clinical and economic evidence  on follow-up strategies for adult cancer patients | (a) Micro  (b) No | CHEERS checklist | Australia, Canada, Finland, Netherlands, Spain, Sweden, UK | (a) Similar trend in CE results  (b) For LMICs  (c) HIC | 5 |
| Browall, 2017^11^ | (a) Medical/ Public Health  (b) Not stated | (a) 2007  (b) World/  Sweden  (c) Cost-of illness  (d) 29 | How interventions using nurse-led follow-up have been evaluated | (a) Micro  (b) No | Not reported | Australia, UK | (a) Not discussed  (b) Limited number of studies  (c) Very low | 3 |
| Kamal, 2017^12^ | (a) Industry  (b) Industry | (a) 2017  (b) World/  USA  (c) Economic  evaluations  (d) 24 | The effect of cancer treatment  on work productivity in patients and their caregivers | (a) Micro  (b) No | Not standardized | Canada, France, Italy, Iran, NL, Sweden, Singapore, UK, USA | (a) High in methods and outcomes  (b) Retrospective design of most of the studies, recall bias  (c) Low | 4 |
| Sun, 2017^13^ | (a) Nursing  (b) Not stated | (a) 2016  (b) World  (c) Empirical  (d) 25 | Outcomes, barriers and facilitators  associated with return to work | (a) Micro  (b) NA | Not reported | Not clear | (a) Study design, data collection, and outcomes  (b) Timeframe  (c) Very low | 2 |
| Barbieri, 2018^14^ | (a) Health Economics  (b) Not stated | (a) 2016  (b) World/  UK  (c) Economic  evaluations  (d) 11 | Quality of the studies and their relevance  to the UK setting | (a) Micro  (b) No | Not clear | Australia, Canada, NL, Spain, UK, USA | (a) Interventions  (b) High  (c) Very low | 2 |

AUD - Australian dollars; AHEI - Australian Health Expenditure Index; BC – breast cancer; CEA – cost-effectiveness analysis; CPI - consumer price index; ER – Estrogen receptor status; GBP - British Pound; ICER - incremental cost-effectiveness ration; LMICs –low and middle income countries; MBC – metastatic breast cancer; NA - not applicable; NCDs - non-communicable diseases; NL – the Netherlands; OECD - the Organisation for Economic Co-operation and Development; PPP - purchasing power parity; RCTs – randomized controlled trials; UK – United Kingdom; USD - American dollars; USA – United States of America.

AMSTAR - AMSTAR quality score. AMSTAR stands for A MeaSurement Tool to Assess systematic Reviews ([**https://amstar.ca**](https://amstar.ca)). The AMSTAR checklist consists of 11 questions, with answers “yes” to each question accounted with a score of one (Appendix ).

^1^ Profile of the department /organization of the corresponding author; ^2^ Year of search/ last evidence included; ^3^ Type and number of cost studies (if multiple site, only the number of studies on breast cancer outcomes is included); ^4^ As presented by the authors; ^5^ Geographical transferability of the results of the systematic review.

**Appendix 4b. Characteristics of the systematic reviews on costs and cost-effectiveness of breast cancer screening**

| **Author (first), year** | **(a) Department ^1^**  **(b) Funding** | **(a) Year^2^**  **(b) Search/ target setting**  **(c) Type^3^**  **(d) N^3^** | **(a) Key review questions**  **(b) Technology and comparator** | **Currency conversion** | **Quality assessment** | **Countries included** | **(a) Variability ^4^**  **(b) Uncertainty^4^**  **(c) Transferability^5^** | **AMSTAR** |
| --- | --- | --- | --- | --- | --- | --- | --- | --- |
| Wagner, 1998^15^ | (a) Public Health  (b) State or non-for profit | (a) 1996  (b) world / USA  (c) RCT  (d) 16 | (a) Effectiveness and cost of mailed patient reminders for MM.  (b) Mailed BCS reminders vs controls. | USD in 1995 using exchange rate and inflation | Not reported | USA, Australia, NZ | (a) Significant even within one country (USA)  (b) Not discussed  (c) Very low | 2 |
| Baxter, 2001^16^ | (a) Medical  (b) State or non-for profit | (a) 2000  (b) world / Canada  (c) Experimental and observational  (d) 3 | (a) Effectiveness of BSE to screen for BC and to provide recommendations for routine teaching of BSE  (b) BSE vs no screening | NA (USD) | Not reported | USA | (a) Only few USA studies are identified  (b) Only few USA studies are identified  (c) USA | 4 |
| Dinnes, 2001^17^ | (a) Health Research  (b) State or non-for profit | (a) 1999  (b) world / UK  (c) No limit  (d) 4 | (a) The effect of double reading of MM in terms of screening accuracy, patient outcomes and costs.  (b) Double reading vs single-reading of MM. | No | Not reported | UK, France, USA | (a) Consistency in incremental costs per additional cancer between the UK and French studies.  (b) High quality studies, questionable transferability to other countries  (c) Very low | 2 |
| Ho, 2002^18^ | (a) HTA  (b) Not stated | (a) 2001  (b) World / Canada  (c) Trials  (d) 17 | (a) Costs and effects of DM and FSM  (b) DM vs FSM | Not clear (USD) | Not reported | Not clear | (a) Not discussed  (b) Not discussed  (c) Very low | 4 |
| Mandelblatt, 2003^19^ | (a) Medical  (b) State or non-for profit | (a) 2002  (b) World /USA  (c) Models  (d) 10 | (a) Costs and benefits of screening women beyond age 65 years.  (b) MM vs screening cessation at 65 years. | USD in 2002 using CPI for medical care | Not reported | USA, NL, UK | (a) Results are stated as "fairly consistent"  (b) Not discussed (sensitivity analysis is used in models)  (c) Very low | 3 |
| Baron, 2008^20^ | (a) Health Marketing  (b) State or non-for profit | (a) 2004  (b) World / USA  (c) No limit  (d) 0 | (a) Efficiency of reducing structural barriers and out-of-pocket costs in increasing BCS.  (b) Client-directed interventions vs no intervention | NA | For economic studies: not reported | NA (no studies included) | (a) NA (no studies included)  (b) NA (no studies included)  (c) NA (no studies included) | 4 |
| Baron, 2010^21^ | (a) Public Health  (b) Mixed | (a) 2004  (b) HICs / USA  (c) No limit  (d) 2 | (a) Effectiveness, applicability, effıciency, barriers, harms or benefıts of provider reminder/recall interventions to increase screening for cancers.  (b) Provider reminders vs no intervention | NA (USD) | For economic studies: not reported | USA | (a) NA (2 study only)  (b) Not discussed  (c) Not clear | 3 |
| Rashidian, 2013^22^ | (a) Health Economic/ Medical Sciences  (b) State or non-for profit | (a) 2010  (b) World / Iran  (c) Models and trials  (d) 26 | (a) Cost-effectiveness of BCS using MM  (b) MM vs no screening | No time conversion, exchange rate to USD | Checklist by Drummond | India, Hong Kong, the USA, the NL, Australia, Finland, Norway, the UK, France, Germany, Switzerland, NZ, Spain, Korea and Slovenia. | (a) Discrepancies in effect estimates, age at screening, and countries of origin  (b) Not discussed  (c) Low | 4 |
| Yoo, 2013^23^ | (a) Health service/ Preventive medicine  (b) Mixed | (a) 2012  (b) world /Asia  (c) No limit  (d) 16 | (a) Cost-effectiveness of MM screening in different incidence rate of Western and Asian countries.  (b) MM vs no screening. | USD in reference year of costs for each of the article | The Quality of Health Economic Studies (QHES) Instrument | US, European Union (6), China, India, S. Korea, Japan | (a) Significant among the studies  (b) Not discussed  (c) Low | 5 |
| Zelle, 2013^24^ | (a) Community care/ Primary care  (b) Not stated | (a) 2013  (b) LMIC  (c) Models and trials  (d) 24 | (a) Economic evidence from LMICs and its methodological quality.  (b) BC control | No | Adapted checklist by Drummond and Jefferson | Asia, Africa, Global, Europe, Latin America (BCS: Brazil, India, Mexico, Turkey, Ghana and Egypt) | (a) In technologies, methods, and results  (b) Poor quality of studies  (c) Low | 4 |
| Koleva-Kolarova, 2015^25^ | (a) Epidemiology  (b) None | (a) 2015(nc)  (b) World  (c) Models  (d) 7 | (a) Bias of simulation models for BCS.  (b) BCS vs no screening | No | Risk of bias (not standardized) | NL, Switzerland, India, Canada, UK, USA, Spain, Germany, Italy, Sweden, France, Australia | (a) High for ICER  (b) Not discussed costs, significant for effects  (c) Low | 4 |
| Li, 2015^26^ | (a) Medical  (b) None | (a) 2013  (b) LMIC  (c) No limit  (d) 5 | (a) Status of MM programs in LMICs and criteria on which this decision can be based.  (b) MM | No | Not reported | USA, UK, India, Brazil | (a) Not discussed (significant by ICERs)  (b) Not discussed costs  (c) Very low | 1 |
| Abdel-Aleem, 2016^27^ | (a) Medical  (b) Not stated | (a) 2015  (b) World  (c) RCTs and non-RCTs  (d) 1 | (a) Impact of mobile clinic services on women’s and children’s health  (b) Mobile clinics | No (USD) | Risk of bias (Cochrane EPOC criteria and 7 criteria for ITS studies). | USA | (a) NA (1 study only)  (b) Not discussed  (c) Not clear | 7 |
| Health Quality Ontario, 2016^28^ | (a) HTA  (b) State or non-for profit | (a) 2015  (b) World / Canada  (c) Not specified  (d) 1 | (a) CE of US as an adjunct to MM compared with MM alone in BCS for women at average and high risk of developing BC  (b) US+MM vs MM | No | Risk of bias (not standardized) | USA | (a) NA (1 study only)  (b) Not discussed for costs, significant for effects  (c) Not clear | 6 |
| Arnold, 2017^29^ | (a) Health Sciences /Health Economics  (b) None | (a) 2017  (b) World  (c) Models  (d) 18 (both general and high-risk population) | (a) Phases of care delivery and  variation in cost and utility parameters in simulation models  for stratified BCS  (b) BCS vs no BCS | USD in 2014 using PPP | Composed tool | Not clear | (a) Homogenous for general risk population  (b) Not discussed  (c) Low | 6 |
| Posso, 2017^30^ | (a) Epdemiology and Public health/ Cochrane center  (b) No | (a) 2017  (b) World  (c) CEA  (d) 2 | (a) Effectiveness and cost-effectiveness of double reading in DM screening  (b) Double vs single reading in MM | USD in 2015 using PPP | CHEERS | Japan, Spain | (a) NA (only 2 studies)  (b) For generalizability  (c) Low | 7 |

CC - currency conversion; CEA – cost-effectiveness analysis; BCS - breast cancer screening; BIA – budget impact analysis; DM and FSM; DR-M – digital radiography mammography; FSM – film-screen mammography; LMICs – low and middle income countries; MM – mammography; RCT – randomized controlled trial; US – ultrasound screening. BC – breast cancer; HIC – high-income countries; ICER – incremental cost-effectiveness ratios; LMICs – low and middle income countries; NA – Not applicable; NL – the Netherlands; NZ – New Zealand; PPP - Purchasing power parity; UK - United Kingdom; USA – United States of America; AMSTAR - quality score. AMSTAR stands for A MeaSurement Tool to Assess systematic Reviews ([**https://amstar.ca**](https://amstar.ca)). The AMSTAR checklist consists of 11 questions, with answers “yes” to each question accounted with a score of one.

**References to the Appendix 4.**

1. Collins RF, Bekker HL, Dodwell DJ. Follow-up care of patients treated for breast cancer: a structured review. *Cancer Treat Rev* 2004;**30**: 19-35.

2. de Boer AG, Taskila T, Ojajarvi A, van Dijk FJ, Verbeek JH. Cancer survivors and unemployment: a meta-analysis and meta-regression. *Jama* 2009;**301**: 753-62.

3. Campbell JD, Ramsey SD. The costs of treating breast cancer in the US: A synthesis of published evidence. *PharmacoEconomics* 2009;**27**: 199-209.

4. Lewis R, Neal RD, Williams NH, France B, Wilkinson C, Hendry M, Russell D, Russell I, Hughes DA, Stuart NS, Weller D. Nurse-led vs. conventional physician-led follow-up for patients with cancer: systematic review. *J Adv Nurs* 2009;**65**: 706-23.

5. Foster TS, Miller JD, Boye ME, Blieden MB, Gidwani R, Russell MW. The economic burden of metastatic breast cancer: A systematic review of literature from developed countries. *Cancer Treatment Reviews* 2011;**37**: 405-15.

6. van Hezewijk M, Elske van den Akker M, van de Velde CJH, Scholten AN, Hille ETM. Costs of different follow-up strategies in early breast cancer: A review of the literature. *Breast* 2012;**21**: 693-700.

7. Jaspers L, Colpani V, Chaker L, van der Lee SJ, Muka T, Imo D, Mendis S, Chowdhury R, Bramer WM, Falla A, Pazoki R, Franco OH. The global impact of non-communicable diseases on households and impoverishment: a systematic review. *European Journal of Epidemiology* 2014;**30**: 163-88.

8. Muka T, Imo D, Jaspers L, Colpani V, Chaker L, van der Lee SJ, Mendis S, Chowdhury R, Bramer WM, Falla A, Pazoki R, Franco OH. The global impact of non-communicable diseases on healthcare spending and national income: a systematic review. *European Journal of Epidemiology* 2015;**30**: 251-77.

9. Chaker L, Falla A, van der Lee SJ, Muka T, Imo D, Jaspers L, Colpani V, Mendis S, Chowdhury R, Bramer WM, Pazoki R, Franco OH. The global impact of non-communicable diseases on macro-economic productivity: a systematic review. *European Journal of Epidemiology* 2015;**30**: 357-95.

10. Meregaglia M, Cairns J. Economic evaluations of follow-up strategies for cancer survivors: A systematic review and quality appraisal of the literature. *Expert Review of Pharmacoeconomics and Outcomes Research* 2015;**15**: 913-29.

11. Browall M, Forsberg C, Wengstrom Y. Assessing patient outcomes and cost-effectiveness of nurse-led follow-up for women with breast cancer - have relevant and sensitive evaluation measures been used? *J Clin Nurs* 2017;**26**: 1770-86.

12. Kamal KM, Covvey JR, Dashputre A, Ghosh S, Shah S, Bhosle M, Zacker C. A systematic review of the effect of cancer treatment on work productivity of patients and caregivers. *Journal of Managed Care and Specialty Pharmacy* 2017;**23**: 136-62.

13. Sun Y, Shigaki CL, Armer JM. Return to work among breast cancer survivors: A literature review. *Supportive Care in Cancer* 2017;**25**: 709-18.

14. Barbieri M, Richardson G, Paisley S. The cost-effectiveness of follow-up strategies after cancer treatment: A systematic literature review. *British Medical Bulletin* 2018;**126**: 85-100.

15. Wagner TH. The effectiveness of mailed patient reminders on mammography screening: A meta-analysis. *American Journal of Preventive Medicine* 1998;**14**: 64-70.

16. Baxter N. Preventive health care, 2001 update: should women be routinely taught breast self-examination to screen for breast cancer? *Cmaj* 2001;**164**: 1837-46.

17. Dinnes J, Moss S, Melia J, Blanks R, Song F, Kleijnen J. Effectiveness and cost-effectiveness of double reading of mammograms in breast cancer screening: Findings of a systematic review. *Breast* 2001;**10**: 455-63.

18. Ho C, Hailey D,, Warburton R, MacGregor J, Pisano E, Joyce J. , Digital mammography versus film-screen mammography: technical, clinical and economic assessments. Technology report no 30. Canadian Coordinating Office for Health Technology Assessment, 2002.

19. Mandelblatt J, Saha S, Teutsch S, Hoerger T, Siu AL, Atkins D, Klein J, Helfand M. The cost-effectiveness of screening mammography beyond age 65 years: a systematic review for the U.S. Preventive Services Task Force. *Ann Intern Med* 2003;**139**: 835-42.

20. Baron RC, Rimer BK, Breslow RA, Coates RJ, Kerner J, Melillo S, Habarta N, Kalra GP, Chattopadhyay S, Wilson KM, Lee NC, Mullen PD, et al. Client-Directed Interventions to Increase Community Demand for Breast, Cervical, and Colorectal Cancer Screening. A Systematic Review. *American Journal of Preventive Medicine* 2008;**35**.

21. Baron RC, Melillo S, Rimer BK, Coates RJ, Kerner J, Habarta N, Chattopadhyay S, Sabatino SA, Elder R, Leeks KJ. Intervention to Increase Recommendation and Delivery of Screening for Breast, Cervical, and Colorectal Cancers by Healthcare Providers. A Systematic Review of Provider Reminders. *American Journal of Preventive Medicine* 2010;**38**: 110-7.

22. Rashidian A, Barfar E, Hosseini H, Nosratnejad S, Barooti E. Cost effectiveness of breast cancer screening using mammography; a systematic review. *Iranian Journal of Public Health* 2013;**42**: 347-57.

23. Yoo KB, Kwon JA, Cho E, Kang MH, Nam JM, Choi KS, Kim EK, Choi YJ, Park EC. Is mammography for breast cancer screening cost-effective in both Western and asian countries?: results of a systematic review. *Asian Pac J Cancer Prev* 2013;**14**: 4141-9.

24. Zelle SG, Baltussen RM. Economic analyses of breast cancer control in low- and middle-income countries: a systematic review. *Syst Rev* 2013;**2**: 20.

25. Koleva-Kolarova RG, Zhan Z, Greuter MJ, Feenstra TL, De Bock GH. Simulation models in population breast cancer screening: A systematic review. *Breast* 2015;**24**: 354-63.

26. Li J, Shao Z. Mammography screening in less developed countries. *Springerplus* 2015;**4**: 615.

27. Abdel-Aleem H, El-Gibaly OM, El-Gazzar AF, Al-Attar GS. Mobile clinics for women's and children's health. *Cochrane Database Syst Rev* 2016: Cd009677.

28. Ontario health technology assessment series ultrasound as an adjunct to mammography for breast cancer screening: A health technology assessment 2016;**16**: 1-71.

29. Arnold M. Simulation modeling for stratified breast cancer screening - a systematic review of cost and quality of life assumptions. *BMC Health Serv Res* 2017;**17**: 802.

30. Posso M, Puig T, Carles M, Rue M, Canelo-Aybar C, Bonfill X. Effectiveness and cost-effectiveness of double reading in digital mammography screening: A systematic review and meta-analysis. *Eur J Radiol* 2017;**96**: 40-9.
