# Supplementary material for: Systematic reviews as a “lens of evidence”: Determinants of cost‐effectiveness of breast cancer screening
Source: Cancer Med. 2019 Sep 30;8(18):7846–58. doi: 10.1002/cam4.2498 (PMC6912065; doi:10.1002/cam4.2498)
Supplement: Supplementary file 5 [file CAM4-8-7846-s005.docx]

# Appendix 5. Outcomes related to breast cancer costs

| **Author, year** | **Outcomes** | **Reported values** | **Reported conclusions on costs or heterogeneity** |
| --- | --- | --- | --- |
| Mandelblatt^23^, 2003 | (a) Diagnostic costs per person (2002)  (b) Treatment costs (2002) | (a) 451-2,520 USD  (b) 7,991 (surgery only) – 63,455 USD |  |
| Collins, 2004^33^ | (a) Follow-up costs  (b) Number of follow up visits | (a) 362- 297 USD (6-18 to 18-30 months after treatment)  (b) 6.3 ( first year) to 3.5 (fourth year) | Lower follow up costs in primary care |
| Boer, 2009^32^ | Unemployment 0.75-9 years after diagnosis (1994 – 2008) | 35.6% vs 31.7% (control);  Pooled RR 1.28 (95% CI, 1.11-1.49) | (a) Lose of unemployment depends on cancer type  (b) Low background overall unemployment rate showed lower risks for unemployment among cancer survivors compared with studies with higher background unemployment rates. |
| Campbell, 2009^35^ | (a) Total BC costs (lifetime, ≤ 2003)  (b) Productivity (wages, 1995)  (c) Dependent care (1997)  (d) Follow up  (e) Adjuvant chemo (2003) | (a) 20-100,000 USD  (b) 4,323-5,900 USD  (c) 43,190 USD  (d) 1,134-1,841 USD  (e) 23,019 – 31,143 USD | (a) Costs increase with initial and terminal than continuing treatment, recurrence of BC than initial BC, higher stages of cancer and younger age of diagnosis.  (b) Direct medical costs were greater than continuing care.  (c) Continuing care accounted largest share of lifetime costs.  (d) Low variability in costs of surgeries  (e) Higher costs for those who received adjuvant chemotherapy;  (f) Highest estimates based on providers charges |
| Lewis, 2009^36^ | Resource use during the follow up, rate per person-year:  (a) Call  (b) Referral to the hospital  (c) Nurse visit  (d) Physician visit  (e) MM  (f) Lab  (g) X-ray  (h) CT  (i) US | (a) 0.03-0.56  (b) 0.09-0.12  (c) 0.03-0.22  (d) 0.96-1.62  (e) 0.80  (f) 0.63  (g) 0.20  (h) 0.18  (i) 0.05 | Lower cost of nurse-led than physician-led follow-up |

| Foster, 2011^37^ | (a) Total per-patient costs of MBC, Sweden (2005)  (b) Informal care, Sweden (2005)  (c) Trastuzumab costs per patient per year (Canada, France, USA, 2005)  (d) Monthly cost of Stage IV BC (UK, 2002)  (e) Mean monthly MBC costs per patient (USA, 1999-2008)$  (f) Indirect costs of early retirement (Sweden, 2005)  (g) National costs for MBC  (1) Discard on dispensed prescriptions on trastuzumab (Australia)  (2) Stage IV BC (UK, 2002)  (3) MBC national trastuzumab costs per year (USA) | (a) 17,031 - 48,169 USD  (b) 8,350 SEK (35% of wages)  (c) 28,000 USD – 28,400 EUR (44% of all cost with other 41% for hospitalization)  (d) 679 GBP (25%) - active treatment and its follow-up, 675 GBP (25%) -supportive care, 1,316 GBP (49%) - end-of-life care  (e) 1,600-5,000 USD (56.4% - cancer  drug, 11.4% - administration, 32.3% other visit-related services), cost variability independent on year of the original evidence  (f) Use of leave or holiday time for caregivers, 54,987 -121,919 SEK  (g) National costs for MBC  (1) 21.1 million USD  (2) 245 million GBP  (3) 370 million USD | (a) Total BC costs much lower for patients >64 y.o., and slightly lower for younger 50 (Sweden), decrease with age (the USA);  (b) Indirect costs higher for those aged 50–64 than younger women.  (c) Discard on dispensed prescriptions for trastuzumab (Australia) |
| --- | --- | --- | --- |
| van Hezewijk, 2012*^38^ | Follow up costs  (a) Annual costs per patient (NL, 2008; Sweden, 2001; UK, 1992, 2009; Spain,1999; Finland, 1995)  (b) Number of visits in 3 years (Spain) | (a) 2975-6192 EUR (NL), 495 – 630 EUR (Sweden), 49 – 146 GBP (UK), 130-426 EUR (Spain), 250-540 EUR (Finland)  (b) 359 - 355 visites | Lower cost with less intensive follow up without impact on survival |
| Jaspers, 2014^39^ | (a) Mean direct OOP medical costs (2013): USA, China (drug)  (b) Mean OOP costs (2013): USA, Australia, Canada  (c) Non-medical costs (2013)  (1) Cost of informal care (USA)  (2) Direct (USA)  (3) Income lost (per year, USA, Canada)  (d) Indirect costs: USA, Australia  (e) Financial hardship: USA, Canada, Pakistan, Norway (income deviation)  (f) Quitting work (NZ) | (a) 8,833 USD (USA); 231-1,028 USD (China)  (b) 4,105-8,833USD (USA), 3,730-5,938 USD (Australia), 4,876 USD (Canada)  (c) Non-medical costs:  (1) 12,693-31,601 USD (first and last 12 months)  (2) 1,938USD  (3) 21,528 USD (USA), 10,101 USD (Canada)  (d) 10,757 USD (USA), 5,036 USD (Australia)  (e) Financial hardship (1-97%)**: 19-80% (USA), 2-15% (Canada), 70% (Pakistan), 6% (Norway)  (f) 27% | OOP and financial household impact may be higher (USA) or lower (China) when population has no societal health insurance. |
| Chaker, 2015^43^ | (a) Unemployment (3-12 months), %  (1) Germany  (2) USA  (3) France  (b) Unemployment (2-3 years), %  (1) NL  (2) France  (3) Denmark  (4) Spain  (c) Unemployment (6-9 years), %  (1) USA  (2) Sweden  (3) Canada  (4) Germany  (d) Temporary absence (to control)  (1) 3 months  (2) 6 months  (3) 3 years  (4) 5 years  (e) Return to work (average)  (1) Sweden  (2) NL  (f) Return to work (in 1 year):  (1) France  (2) USA  (g) Productivity loss costs (the USA) | (a) Unemployment (3-12 months)  (1) 49.8% (3 time more the general population)  (2) 26-52%  (3) 54%  (b) Unemployment (2-3 years)  (1) 11%  (2) 18%  (3) 28%  (4) 46 %  (c) Unemployment (6-9 years), %  (1) 18%  (2) 26%  (3) 41%  (4) 43%  (d) Temporary absence (since diagnosis)  (1) 26-40% (USA)  (2) 18%  (3) 11%  (4) 10% (USA, Sweden)  (e) Return to work (average), months  (1) 3  (2) 11.4  (f) Return to work (in 1 year):  (1) 54.3%  (2) 82%  (g) 5.5 – 20.9 billion USD | 1. Productivity costs and return to work within 3 month since diagnosis is higher for white than for black women; 2. Unemployment rate Is the highest during the second year; 3. Return-to-work vary significantly by country |
| Meregaglia, 2015^31^ | Follow-up:   1. Australia (2013) 2. Spain (2013) 3. Canada (30 days, 2013) 4. Sweden (2009) 5. UK (2009) | (a) 407–4,132 AUD  (b) 23-37 EUR  (c) 136 -381 USD  (d) 495-630 EUR  (e) 62-90 GBP | Lower cost and favourable cost-effectiveness with less intensive follow up (frequency, length, risk adapted by age and tumor stage) in primary care or through mobile-app technologies vs hospital-based investigations. |
| Muka, 2015^42^ | (a) Mean annual total direct costs (2013)  (b) Total costs (Belgium, USA, 2013)  (c) Mean costs for metastatic BC (USA, 2013)  (d) Mean indirect costs (2013)  (e) Annual worldwide economic burden | (a) 4,595 - 82,794 USD  (b) 30,000 USD  (c) 105,310 USD  (d) 2,109 - 24,740 USD  (e) 30.9 billion USD (46% -medication, 27% non-medical, 27% - productivity) | 1) Annual direct costs of NCDs were the highest in the Americas  (2) BC treatment costs 52 % higher for stage II compared to 0;  (3) Higher costs of metastiatic BC |
| Browall, 2016^26^ | Follow up  (a) Difference in travel and productivity costs between hospital and phone follow up (UK, 2008) up to 24m  (b) Hospital follow up plus educational program (2011) | (a) 40 – 55 GBP  (b) 4,914 EUR | Higher follow up costs in hospital than nurse phone follow up (though increase in number of calls and higher patient satisfaction)  Nurse-led telephone follow-up plus educational program could decrease costs while increasing quality adjusted life years. |
| Arnold, 2017^45^ | (a) Cost of recall (2014)  (b) Biopsy (2014)  (c) BC in situ costs  (d) Initial treatment  (1) Local  (2) Regional  (3) Metastatic  (e) End of life  (1) Local  (2) Regional  (3) Metastatic | (a) 65 - 442USD  (b) 290-1,700USD  (c) 8,088-13,696USD  (d) Initial treatment  (1) 10,650-15,239USD  (2) 13,042-25,894USD  (3) 28,239 – 42,115USD  (e) End of life  (1) 16,939-35,300USD  (2) 23,003-43,879USD  (3) 21,089 – 61,545 USD | Higher costs of end of life than initial treatment and for metastatic than local or region |
| Kamal, 2017^20^ | Productivity  (a) Temporary absence  (1) missed ≥1 month of work (NL, USA)  (2) missed ≥1 year of work (NL)  (3) quit job at diagnosis (USA, Italy, Iran)  (4) mean duration of sick leave (France, Sweden, Iran), months  (b) Unemployment (3 month - 2 years)  (1) Sweden  (2) Italy  (b) Unemployment (2-3 years)  (1) NL  (2) France  (c) Return to work (%)  (1) No interruption (France)  (2) 6 months (Sweden)  (3) 12 months (France)  (4) 3 years (France) | Productivity  (a) Temporary absence  (1) 85% NL, 24% USA  (2) 35%  (3) 32% -USA, 45% - Italy, 20% - Iran  (4) 10.8 - France, 13.7 – Sweden, 3.5 - Iran  (b) Unemployment (3 month - 2 years)  (1) 20%  (2) 55%  (b) Unemployment (2-3 years)  (1) 6%  (2) 18%  (c) Return to work (%)  (1) 31%  (2) 28-85% (depending if received chemotherapy)  (3) 54%  (4) 82% | (a) Factors negatively impacting productivity: disease progression or severity, treatment-related side effects that led to missed work, treatment-associated cognitive impairments, and lifting heavy loads and keeping pace with others at work.  (b) Greater limitations after return to work, longer periods of sick leave or delayed return, and greater probability of not returning to work with chemotherapy.   1. Cumulative effects of multimodal treatments. |
| Sun, 2017^47^ | (a) Return to work timeframe  (1) <6 weeks  (2) 1 year  (3) 1- 3 years  (4) >=3 year  (b) Mean delay in return to work | (a) Return to work timeframe  (1) 20-44%  (2) 59%  (3) 56 -65%  (4) 82%  (b) 10.8 - 11.4 months | Factors impacting productivity: Health and well-being (overall well-being, satisfaction with daily living), Symptoms and function (anxiety, fatigue, depressive symptoms), Work demands and work environment (flexibility, demand, work-related stress, work support), Individual characteristics (coping skills, personality, disease stage), Societal and culture factors (social coping resources, advices). |
| Barbieri, 2018^18^ | Follow up  (a) Total cost difference between phone and hospital (UK), primary and secondary (Spain), not intensive and intensive (Spain, Australia), mobile versus in-person (Canada) per patient per year  (b) Difference in travel and productivity costs between hospital and phone follow up (UK, 2008)  (c) Preferences to secondary/ primary /or any follow up. | (a) 55 GBP (UK), 72 -296 EUR (Spain), 555 AUD (Australia), 245 CAD (Canada)  (b) 47 GBP  (c) 80%/10%/10% | The costs of follow-up in primary care were lower, intensive follow up (additional US, annual mammography) may have no more benefits with higher costs |
| Legend to the Table 2. * From trials only; ** Depends on how it is measured; $ recalculated to per-year. Abbreviations: AUD - Australian dollar; BC – breast cancer; CT -computed tomography scan ; EUR – Euro; GBP - Great British Pound; MBC – metastatic breast cancer; MM – mammography; NCD – non-communicable disease; NL – the Netherlands; NZ – New Zealand; OOP – out-of-pocket payment; RR – relative risk; SEK - Swedish krona; US – ultrasonography, USD - United States dollar; UK – the United Kingdom; y.o. – years old. | | | |
