# Supplementary material for: Systematic reviews as a “lens of evidence”: Determinants of cost‐effectiveness of breast cancer screening
Source: Cancer Med. 2019 Sep 30;8(18):7846–58. doi: 10.1002/cam4.2498 (PMC6912065; doi:10.1002/cam4.2498)
Supplement: Supplementary file 6 [file CAM4-8-7846-s006.docx]

**Appendix 6. Assessment of risk in publication bias in reviews on cost-effectiveness of mammography screening**

**Appendix 6A. Comparison of methods in four reviews assessing cost-effectiveness of mammography screening in comparison to no screening**

|  | **Rashidian, 2013** | **Yoo, 2013** | **Zelle, 2013** | **Koleva-Kolarova, 2015** |
| --- | --- | --- | --- | --- |
| Assessed quality | Yes | Yes | Yes | Yes |
| Included quality in conclusions | No | Yes | Yes | Yes |
| Cost conversion | conversion was made based on the year the study was accepted for publication, USD | USA dollars on the reference year of cost of each article | No | Not for the reported values |
| Year conversion | Not mentioned | Not mentioned | No | Not for the reported values |
| Quality score of the review | 4 | 5 | 4 | 4 |
| Aim | Provide a summary of the best available evidence to support policy makers, especially in countries where local evidence does not suffice, in making informed decision about conducting breast cancer screening | Determine whether mammography mass screening is cost-effective for both Western and Asian countries. | Present the available economic evidence from LMICs and to assess the methodological quality of the analyses. | Critically evaluate published models |
| Specific inclusion criteria | Cost-effectiveness studies alongside randomized controlled trials, or used modelling techniques to estimate cost-effectiveness ratios. | Cost-effectiveness analysis of a screening strategy compared with no screening | Breast cancer, Low-income countries | Multi-use simulation models (not on "population subgroups") |
| Search period | January 1993 -March 2010 | 1974 -June 25, 2012 | Up to in January 2013, | Not clear |
| Language | English | English | English | No limit |
| Conclusions of cost-effectiveness of MM | Biennial MM for 50-70 yo is the most cost-effective option. More studies for LMICs are required. Screening for younger age is not recommended | Cost-effective in most of Western countries, but not in Asian countries. | Little evidence to provide specific recommendations on screening by mammography versus clinical breast examination. | Cost-effective in general, in Europe more than in the USA and India. |

MM – mammography , LMICs – low and middle income countries, USA – United States of America

**Appendix 6B. Comparison in reported incremental cost-effectiveness ratios from four reviews assessing cost-effectiveness of mammography screening in comparison to no screening**

| **Author/year of the original evidence** | **Rashidian, 2013** | **Yoo, 2013** | **Zelle, 2013** | **Koleva-Kolarova, 2015** | **Country*** | **Unit** | **Interval** | **Age** |
| --- | --- | --- | --- | --- | --- | --- | --- | --- |
| Van Ineveld et al., 1993 | $3162 |  |  | EUR 1800-9700 per not clear outcome, not clear program | Netherlands | Cost per LYG | Biennial | 50-70 years |
|  | $2685 |  |  |  | UK | Cost per LYG | Biennial | 50-70 years |
|  | $8651 |  |  |  | France | Cost per LYG | Biennial | 50-70 years |
|  | $14468 |  |  |  | Spain | Cost per LYG | Biennial | 50-70 years |
| Carter et al., 1993 | $14733 |  |  | AUS$13000-27000 per not clear outcome not clear program | Australia | Cost per LYG | Biennial | 50-69 years |
|  | $13081 |  |  |  | Australia | Cost per LYG | Triennial | 50-69 years |
| Rosenquist et al., 1994 | $15,500 |  |  |  | USA | Cost per LYG | Annual | 60-69 years |
| Szeto et al., 1996 | $14510 |  |  | AUS$13000-22000 per not clear outcome | New Zealand | Cost per LYG | Biennial | 50-64 years |
|  | $12668 |  |  |  | New Zealand | Cost per LYG | Triennial | 50-64 years |
|  | $14,597 |  |  |  | New Zealand | Cost per LYG | Biennial | 50-69 years |
| Plans et al., 1996 | $8424 |  |  |  | Spain | Cost per cancer detected | interval not determined | 50-64 years |
| Hakama et al., 1997 | $77100 |  |  |  | Finland | Cost per death averted | Biennial | 50-69 years |
|  | $15400 |  |  |  | Finland | Cost per LYG | Biennial | 50-69 years |
|  | $15900 |  |  |  | Finland | Cost per QALY gained | Biennial | 50-69 years |
| Salzmann et al., 1997 | $45700 |  |  | $21000-168000 per not clear outcome not clear program | USA | Cost per LYG | Annual | 50-69 years |
|  | $46500 |  |  |  | USA | Cost per QALY | Annual | 50-69 years |
|  | $21400 |  |  |  | USA | Cost per LYG | Biennial | 50-69 years |
|  | $21700 |  |  |  | USA | Cost per QALY | Biennial | 50-69 years |
| Boer et al., 1998 | $4195 |  |  | £2500-2,700 not clear outcomes, not clear characteristics | UK | Cost per LYG | Triennial | 50-64 years |
|  | $4343 |  |  |  | UK | Cost per LYG | Triennial | 50-69 years |
|  | $4506 |  |  |  | UK | Cost per LYG | Biennial | 50-64 years |
|  | $41824 |  |  |  | UK | Cost per death averted | Triennial | 50-64 years |
|  | $40265 |  |  |  | UK | Cost per death averted | Triennial | 50-69 years |
|  | $46353 |  |  |  | UK | Cost per death averted | Biennial | 50-64 years |
| Boer et al., 1999 |  |  |  | $8,300-38,600 per not clear outcome | USA | Not clear |  |  |
| Leivo et al., 1999 | $18955 | $18955 |  |  | Finland | Cost per LYG | Biennial | 50-59 years |
| Norum, 1999 | $17202 |  |  |  | Norway | Cost per cancer detected | Biennial | 50-69 years |
|  | $14208 | $14554 |  |  | Norway | Cost per LYG | Biennial | 50-69 years |
| Wang et al., 2001 | $3750 |  |  |  | Norway | Cost per LYG | Biennial | 50-69 years |
|  | $86045 |  |  |  | Norway | Cost per life saved | Biennial | 50-69 years |
| Arveux et al., 2003 | $25000 |  |  |  | France | Cost per LYG | Annual | 50-65 years |
| Groot et al., 2006 | $75 |  | MM screening is cost-effective (quality 86%) |  | Africa | Cost per DALY averted | Biennial | 50-70 years |
|  | $915 |  |  |  | North America | Cost per DALY averted | Biennial | 50-70 years |
|  | $75 |  |  |  | Asia | Cost per DALY averted | Biennial | 50-70 years |
| Stout et al., 2006 | $27000 | $27000 |  | $27000-58000 per not clear outcome, not clear program | USA | Cost per QALY gained | 5-yearly | 55-70 years |
|  |  | $28000 |  |  | USA | Cost per QALY gained or LYS | Triennial | 55-70 years |
|  |  | $31000 |  |  | USA | Cost per QALY gained or LYS | Triennial | 50-75 years |
|  |  | $31000 |  |  | USA | Cost per QALY gained or LYS | Triennial | 45-75 years |
|  |  | $34000 |  |  | USA | Cost per QALY gained or LYS | Biennial | 50 -75 years |
|  |  | $34000 |  |  | USA | Cost per QALY gained or LYS | Biennial | 45 -75 years |
|  |  | $47000 |  |  | USA | Cost per QALY gained or LYS | Biennial | 40 -80 years |
|  |  | $49000 |  |  | USA | Cost per QALY gained or LYS | Annual | 45 -75 years |
|  |  | $53000 |  |  | USA | Cost per QALY gained or LYS | Annual | 45 -80 years |
|  |  | $58000 |  |  | USA | Cost per QALY gained or LYS | Annual | 40 -81 years |
| Rojnik et al., 2008 | $9801 |  |  |  | Slovenia | Cost per QALY gained | Triennial | 50-65 years |
| de Gelder et al., 2009 | $16895 |  |  | EUR 11000-28000 cost per effect (not precised) | Switzerland | Cost per LYG | Biennial | 50-69 years |
|  | $18233 |  |  |  | Switzerland | Cost per QALY gained | Biennial | 50-69 years |
|  |  | $15486-31732 |  |  | Switzerland | Cost per QALY gained or LYS | Biennial | 50-69 years |
|  |  | $34375 |  |  | Switzerland | Cost per QALY gained or LYS | Annual/Biennial | 50-69 years |
| Rosenquist et al., 1994 | $35000 |  |  |  | USA | Cost per LYG | Annual | 80-85 years |
|  | $26200 |  |  |  | USA | Cost per LYG | Annual | 40-49 years |
|  | $14000 |  |  |  | USA | Cost per LYG | Biennial | 40-49 years |
| Madan et al., 2010 | $44692 |  |  | £ 17000-30000 per not clear outcome | UK | Cost per QALY gained | Triennial | 47-49 years |
| Hall et al., 1992 | $7190 |  |  |  | Australia | Cost per LYG | Biennial | 45-69 years |
| Carter et al., 1993 | $ 27257 |  |  |  | Australia | Cost per LYG | Annual 40-49 plus biennial 50-69 | |
|  | $19919 |  |  |  | Australia | Cost per LYG | Biennial 40-49 plus triennial 50-69 | |
|  | $20300 |  |  |  | Australia | Cost per LYG | Biennial | 40-49 years |
| Rosenquist et al., 1994 | $18600 |  |  |  | USA | Cost per LYG | Annual | 40-85 years |
|  | $16800 |  |  |  | USA | Cost per LYG | Annual | 50-85 years |
| Lindfors et al., 1995 | $16000-$31900 |  |  | $16000-32000 | USA | Cost per LYG | Different 7 scenarios | min -max ratio |
| Szeto et al., 1996 | $15169 |  |  |  | New Zealand | Cost per LYG | Biennial | 45-64 years |
| Rosenquist et al., 1998 | $16100 -$18800 | $16100 -$18800 |  | $10000-27000 per not clear outcome not clear program | USA | Cost per LYG | Four different age group scenarios: 40-79/1 40-79/1,50-79/1,40-49/2 40-64/1,65-79/2 40-49/1,50-79/2 | min ratio |
| Woo et al., 2007 | $90771 | $90771 |  |  | Hong Kong | Cost per LYG | Biennial | 50-74 years |
|  | $107310 | $107310 |  |  | Hong Kong | Cost per LYG | Biennial | 40- 74 years |
|  | $321608 | $321608 |  |  | Hong Kong | Cost per LYG | Annual | 50 -74 years |
|  | $385092 | $385092 |  |  | Hong Kong | Cost per DALY averted | Annual | 40 -74 years |
| Woo et al., 2007 |  | $130771 |  | $90000-2897 per not clear outcome, not clear program | China (Hong Kong) | Cost per QALY gained or LYS | 3/4 years | 40-49 years |
|  |  | $37101 |  |  | China (Hong Kong) | Cost per QALY gained or LYS | 3/4 years | 50-59 years |
|  |  | $17062 |  |  | China (Hong Kong) | Cost per QALY gained or LYS | 3/4 years | 60-69 years |
|  |  | $10603 |  |  | China (Hong Kong) | Cost per QALY gained or LYS | 3/4 years | 70-79 years |
| Wong et al., 2007 | $64400 | a |  | $62000-179000 per not clear outcome, not clear program | Hong Kong | Cost per LYG | Biennial | 40 -69 years |
|  | $61600 |  |  |  | Hong Kong | Cost per QALY gained | Biennial | 41 -69 years |
| Wong et al., 2010 |  | Dominated |  |  | China (Hong Kong) | Cost per QALY gained or LYS | Biennial | 50-69 years |
|  |  | Dominated |  |  | China (Hong Kong) | Cost per QALY gained or LYS | Biennial | 50-79 years |
|  |  | $64400 |  |  | China (Hong Kong) | Cost per QALY gained or LYS | Biennial | 40 -69 years |
|  |  | $206300 |  |  | China (Hong Kong) | Cost per QALY gained or LYS | Biennial | 40 -79 years |
|  |  | $37000 |  |  | USA | Cost per QALY gained or LYS | Biennial | 50-69 years |
|  |  | Dominated |  |  | USA | Cost per QALY gained or LYS | Biennial | 50-79 years |
|  |  | $47800 |  |  | USA | Cost per QALY gained or LYS | Biennial | 40 -69 years |
|  |  | $80400 |  |  | USA | Cost per QALY gained or LYS | Biennial | 40 -79 years |
| Okonkwo et al., 2008 | $6496 | $1634 | “CBE screening in India compares favourably with MM screening in developed countries (quality 84%)” | $3000-19000 per not precise currency unit | India | Cost per LYG | Once | 40 aged |
|  | $1634 | $6496 |  |  | India | Cost per QALY gained or LYS | Once | 50 aged |
|  | $3468 | $3468 |  |  | India | Cost per LYG | Biennial | 40-60 years |
|  | $110542 |  |  |  | India | Cost per death averted | Once | 40 aged |
|  | $46021 |  |  |  | India | Cost per death averted | Biennial | 40-60 years |
|  |  |  |  |  | India | Cost per QALY gained or LYS | Biennial | 40-70 years |
|  | $3308 | $3308 |  |  | India | Cost per LYG | Biennial | 50-70 years |
|  | $22220 |  |  |  | India | Cost per death averted | Once | at age 50 |
|  | $36731 |  |  |  | India | Cost per death averted | Biennial | 50-70 years |
| Lee et al., 2009 | $100007 | a |  |  | Korea | Per cancer detected | Triennial | 45-65 years |
| Knox, 1998 |  | $3730 |  | £3000-34000 | UK | Cost per QALY gained or LYS | Triennial | 50-65 years |
| Van der Maas, 1989 |  | $4050 |  |  | Netherlands | Cost per QALY gained or LYS | 4 years | 52 - 68 years |
|  |  | $4850 |  |  | Netherlands | Cost per QALY gained or LYS | Biennial | 51 - 69 years |
|  |  | $5900 |  |  | Netherlands | Cost per QALY gained or LYS | 3-4 years | 50 - 69 years |
|  |  | $6840 |  |  | Netherlands | Cost per QALY gained or LYS | Annual | 50 -69 years |
| Okubo, 1991 |  | $14300 |  | $14000-40000 per not clear outcome | Japan | Cost per QALY gained or LYS | Annual | 30 -80 years |
| Garuz, 1997 |  | $8833 |  | European currency unit 2100-7300 per not clear outcome | Spain | Cost per QALY gained or LYS | Biennial | 50 -65 years |
|  |  | $11374 |  |  | Spain | Cost per QALY gained or LYS | Biennial | 45-65 years |
| Carles, 2011 |  | $4691 |  | EUR 3000 - 725000 per not clear outcome not clear program | Spain | Cost per QALY gained or LYS | Biennial | 50 -69 years |
|  |  | $9555 |  |  | Spain | Cost per QALY gained or LYS | Biennial | 45-65 years |
|  |  | $16041 |  |  | Spain | Cost per QALY gained or LYS | Annual | 45 -69 years |
|  |  | $22020 |  |  | Spain | Cost per QALY gained or LYS | Annual | 40 -69 years |
|  |  | $33256 |  |  | Spain | Cost per QALY gained or LYS | Annual | 40 -74 years |
|  |  | $614811 |  |  | Spain | Cost per QALY gained or LYS | Annual | 40 -79 years |
| Koning, 1991 |  | $3825 |  | $3000-6000 per not clear outcome not clear program | Netherlands | Cost per QALY gained or LYS | Biennial | 50 -70 years |
|  |  | $5385 |  |  | Netherlands | Cost per QALY gained or LYS | Biennial | 40 -70 years |
|  |  | $4670 |  |  | Netherlands | Cost per QALY gained or LYS | 1.3 years | 50 -70 years |
|  |  | $4100 |  |  | Netherlands | Cost per QALY gained or LYS | Biennial | 50 -75 years |
|  |  | $3235 |  |  | Netherlands | Cost per QALY gained or LYS | Triennial | 50 -65 years |
| Kang, 2013 |  | $29964 |  |  | South Korea | Cost per QALY gained or LYS | Biennial | 40- years |
| Sarvazyan, 2008 |  | a | Tactile imaging has the potential to provide cost-effective breast cancer screening and diagnosis, quality 45% |  | Worldwide | Life years saved | not clear | not reported |
| Ginsberg, 2012 |  | a | I$2,248 -4,596, cost-effective (quality 90%) |  | Cameroon | Cost per DALY averted | not clear | not reported |
| Salomon, 2012 |  | a | I$22000, potentially cost-effective (quality 78%) |  | Mexico | Cost per DALY averted | not clear | not reported |
| Szynglarewicz and Matkowski, 2011 |  | a | Cost-effective even for middle-income countries (quality 33%) |  | Poland | not reported | not reported | not reported |
| Yazihan and Yilmaz, 2006 |  | a | Cost-effective (quality 40%) |  | Turkey | Cost per DALY averted | not reported | not reported |
| Astim, 2011 |  | a | Cost-effective (biennial, 40+), quality 57% |  | Turkey | Intermediate outcome measures | Biennial, Annual | (40+, 45+, 50+, 55+, 60+ years |
| Zelle, 2012 |  |  | Not cost-effective (quality 90%) |  | Ghana | Cost per DALY averted |  |  |
| Mousavi, 2008 |  |  | No clear conclusion (quality 23%) |  | Iran | Life years saved |  | 35-69, 50-69 years |
| Warmerdam, 1997 |  |  |  | German Mark 15-24000 per not clear outcome, not clear program | Germany |  |  |  |
| Van den Akker -van Marie, 1997 |  |  |  | 427000-555000 Spanish pesto per not clear outcome, not clear program | Spain |  |  |  |
| Vanara, 1997 |  |  |  | EUR 6587 - 11518 per not clear outcome, not clear program | Italy |  |  |  |
| Boer, 1995 |  |  |  | £8400-36000 per not clear outcome, not clear program | Netherlands |  |  |  |
| Beemsterboer, 1994 | $11135 per LYG - 11725 (per QALY) |  |  | German Mark 18800-25300 per not clear outcome, not clear program | Germany |  | Biennial | 50-69 years |
| Mandelblatt, 2005 |  |  |  | $58000-151000 (2005),per not clear outcome, not clear program | USA |  |  |  |
| Mandelblatt, 2004 |  |  |  | $53000-124000 (2004) per not clear outcome, not clear program | USA |  |  |  |
| Tosteson, 2008 |  |  |  | $26500-272000 (2008), $27000-58000 (2006) per not clear outcome, not clear program | USA |  |  |  |
| Peregrino, 2012 |  |  |  | $13600-2904 (?) per not clear outcome, not clear program | Brazil |  |  |  |
| Zhang, 2012 |  |  |  | Chinese yuan 217000-248000 per not clear outcome, not clear program | China |  |  |  |
| Wong, 2012 |  |  |  | $73000-204000 | Hong Kong (China) | |  |  |
| Valencia-mendoza, 2009 |  |  |  | Mexican pesos 75300-171100 per not clear outcome, not clear program | Mexico |  |  |  |
| Sato, 2014 |  |  |  | Japanese Yen 310800 per not clear outcome, not clear program | Japan |  |  |  |
| Rojnik, 2008 |  |  |  | EUR 4000-42000 per not clear outcome | Slovenia |  |  |  |
| Neeser, 2007 |  |  |  | $73000-118000 per not clear outcome | Switzerland |  |  |  |
| Melnikow, 2013 |  |  |  | $17000-18000 per not clear outcome, not clear program | USA |  |  |  |
| Schousboe, 2011 |  |  |  | $8000-363000 per not clear outcome, not clear program | USA |  |  |  |
| Ahern, 2009 |  |  |  | $36000-3939 per not clear outcomes, not clear program | USA |  |  |  |
| Carter, 2005 |  |  |  | $9000-580000 per not clear outcome not clear program | USA |  |  |  |
| Shen, 2005 |  |  |  | $49000-92000 per not clear outcome not clear program | USA |  |  |  |
| Michaelson, 1999 |  |  |  | $5000-1019 per not clear outcome not clear program | USA |  |  |  |

CBE – Clinical breast examination, DALY – disability adjusted life year, LYG – life year gained, LYS – life-year saved, MM – mammography screening, UK – United Kingdom, USA – United States of America, QALY – quality adjusted life years.

Currency abbreviations: AU$ - Australian dollars. $ - American dollar, £ - British pound.

**Appendix 6c. Cost-effectiveness of breast cancer screening programs in economic evaluations included in the reviews on cost-effectiveness of mammography screening**

| **Author/year of the original evidence** | **Values, $** | **GDP –ICER, $** | **3GDP –ICER, $** | **Country** |
| --- | --- | --- | --- | --- |
| Van Ineveld et al., 1993 | 3162 | 20437 | 67635 | Netherlands |
|  | 2685 | 17802 | 58777 | UK |
|  | 8651 | 15163 | 62790 | France |
|  | 14468 | 1600 | 33737 | Spain |
| Carter et al., 1993 | 14733 | 3837 | 40976 | Australia |
|  | 13081 | 5489 | 42628 | Australia |
| Rosenquist et al., 1994 | 15500 | 10965 | 63895 | USA |
| Szeto et al., 1996 | 14510 | 2890 | 37691 | New Zealand |
|  | 12668 | 4732 | 39533 | New Zealand |
|  | 14597 | 2803 | 37604 | New Zealand |
| Plans et al., 1996 | 8424 | 7006 | 37866 | Spain |
| Hakama et al., 1997 | 77100 | -51322 | 233 | Finland |
|  | 15400 | 10378 | 61933 | Finland |
|  | 15900 | 9878 | 61433 | Finland |
| Salzmann et al., 1997 | 45700 | -15632 | 44505 | USA |
|  | 46500 | -16432 | 43705 | USA |
|  | 21400 | 8668 | 68805 | USA |
|  | 21700 | 8368 | 68505 | USA |
| Boer et al., 1998 | 4195 | 22426 | 75669 | UK |
|  | 4343 | 22278 | 75521 | UK |
|  | 4506 | 22115 | 75358 | UK |
|  | 41824 | -15203 | 38040 | UK |
|  | 40265 | -13644 | 39599 | UK |
|  | 46353 | -19732 | 33511 | UK |
| Boer et al., 1999 |  | 34621 | 103863 | USA |
| Leivo et al., 1999 | 18955 | 6823 | 58378 | Finland |
| Norum, 1999 | 17202 | 17587 | 87164 | Norway |
|  | 14208 | 20581 | 90158 | Norway |
| Wang et al., 2001 | 3750 | 34397 | 110690 | Norway |
|  | 86045 | -47898 | 28395 | Norway |
| Arveux et al., 2003 | 25000 | -2566 | 42301 | France |
| Stout et al., 2006 | 27000 | 19437 | 112311 | USA |
|  | 28000 | 18437 | 111311 | USA |
|  | 31000 | 15437 | 108311 | USA |
|  | 31000 | 15437 | 108311 | USA |
|  | 34000 | 12437 | 105311 | USA |
|  | 34000 | 12437 | 105311 | USA |
|  | 47000 | -563 | 92311 | USA |
|  | 49000 | -2563 | 90311 | USA |
|  | 53000 | -6563 | 86311 | USA |
|  | 58000 | -11563 | 81311 | USA |
| Rojnik et al., 2008 | 9801 | 14040 | 61723 | Slovenia |
| de Gelder et al., 2009 | 16895 | 55593 | 200569 | Switzerland |
|  | 18233 | 54255 | 199231 | Switzerland |
| Rosenquist et al., 1994 | 35000 | -8535 | 44395 | USA |
|  | 26200 | 265 | 53195 | USA |
|  | 14000 | 12465 | 65395 | USA |
| Madan et al., 2010 | 44692 | -6430 | 70095 | UK |
| Hall et al., 1992 | 7190 | 11634 | 49283 | Australia |
| Carter et al., 1993 | 27257 | -8687 | 28452 | Australia |
|  | 19919 | -1349 | 35790 | Australia |
|  | 20300 | -1730 | 35409 | Australia |
| Rosenquist et al., 1994 | 18600 | 7865 | 60795 | USA |
|  | 16800 | 9665 | 62595 | USA |
| Lindfors et al., 1995 | 16000 | 11777 | 67330 | USA |
|  | 31900 | -4122 | 51433 | USA |
| Szeto et al., 1996 | 15169 | 2231 | 37032 | New Zealand |
| Rosenquist et al., 1998 | 16100 | 15473 | 78618 | USA |
|  | 18800 | 12774 | 75921 | USA |
| Woo et al., 2007 | 90771 | -62547 | -6098 | Hong Kong |
|  | 107310 | -79086 | -22637 | Hong Kong |
|  | 321608 | -293384 | -236935 | Hong Kong |
|  | 385092 | -356868 | -300419 | Hong Kong |
| Woo et al., 2007 | 130771 | -102547 | -46098 | China (Hong Kong?) |
|  | 37101 | -8877 | 47572 | China (Hong Kong?) |
|  | 17062 | 11162 | 67611 | China (Hong Kong?) |
|  | 10603 | 17621 | 74070 | China (Hong Kong?) |
| Wong et al., 2007 | 64400 | -36176 | 20273 | Hong Kong |
|  | 61600 | -33376 | 23073 | Hong Kong |
| Wong et al., 2010 | Dominated |  |  | China (Hong Kong?) |
|  | Dominated |  |  | China (Hong Kong?) |
|  | 64400 | -33703 | 27692 | China (Hong Kong?) |
|  | 206300 | -175603 | -114208 | China (Hong Kong?) |
|  | 37000 | 11375 | 108126 | USA |
|  | Dominated |  |  | USA |
|  | 47800 | 575 | 97326 | USA |
|  | 80400 | -32025 | 64726 | USA |
| Okonkwo et al., 2008 | 6496 | -5478 | -3442 | India |
|  | 1634 | -616 | 1420 | India |
|  | 3468 | -2450 | -414 | India |
|  | 110542 | -109524 | -107488 | India |
|  | 46021 | -45003 | -42967 | India |
|  |  | 1018 | 3054 | India |
|  | 3308 | -2290 | -254 | India |
|  | 22220 | -21202 | -19166 | India |
|  | 36731 | -35713 | -33677 | India |
| Lee et al., 2009 | 100007 | -79119 | -37342 | South Korea |
| Knox, 1998 | 3730 | 22891 | 76134 | UK |
| Van der Maas, 1989 | 4050 | 13126 | 47477 | Netherlands |
|  | 4850 | 12326 | 46677 | Netherlands |
|  | 5900 | 11276 | 45627 | Netherlands |
|  | 6840 | 10336 | 44687 | Netherlands |
| Okubo, 1991 | 14300 | 11059 | 61778 | Japan |
| Garuz, 1997 | 8833 | 7236 | 39375 | Spain |
|  | 11374 | 4695 | 36834 | Spain |
| Carles, 2011 | 4691 | 27643 | 92311 | Spain |
|  | 9555 | 22779 | 87447 | Spain |
|  | 16041 | 16293 | 80961 | Spain |
|  | 22020 | 10314 | 74982 | Spain |
|  | 33256 | -922 | 63746 | Spain |
|  | 614811 | -582477 | -517809 | Spain |
| Koning, 1991 | 3825 | 17630 | 60540 | Netherlands |
|  | 5385 | 16070 | 58980 | Netherlands |
|  | 4670 | 16785 | 59695 | Netherlands |
|  | 4100 | 17355 | 60265 | Netherlands |
|  | 3235 | 18220 | 61130 | Netherlands |
| Kang, 2013 | 29964 | -5605 | 43112 | South korea |
| Ginsberg, 2012 | 2248 | -867 | 1894 | Cameroon |
|  | 4596 | -3215 | -454 | Cameroon |
| Salomon, 2012 | 22000 | -12060 | 7821 | Mexico |
| Beemsterboer, 1994 | 11135 | 15953 | 70128 | Germany |

GDP – gross domestic product per capita; ICER – incremental cost-effectiveness ratio.

**Appendix 6d. Difference between gross domestic product per capita and incremental cost-effectiveness ratio (biannual or triennial screening programs for women starting screening at age 50 or above)**


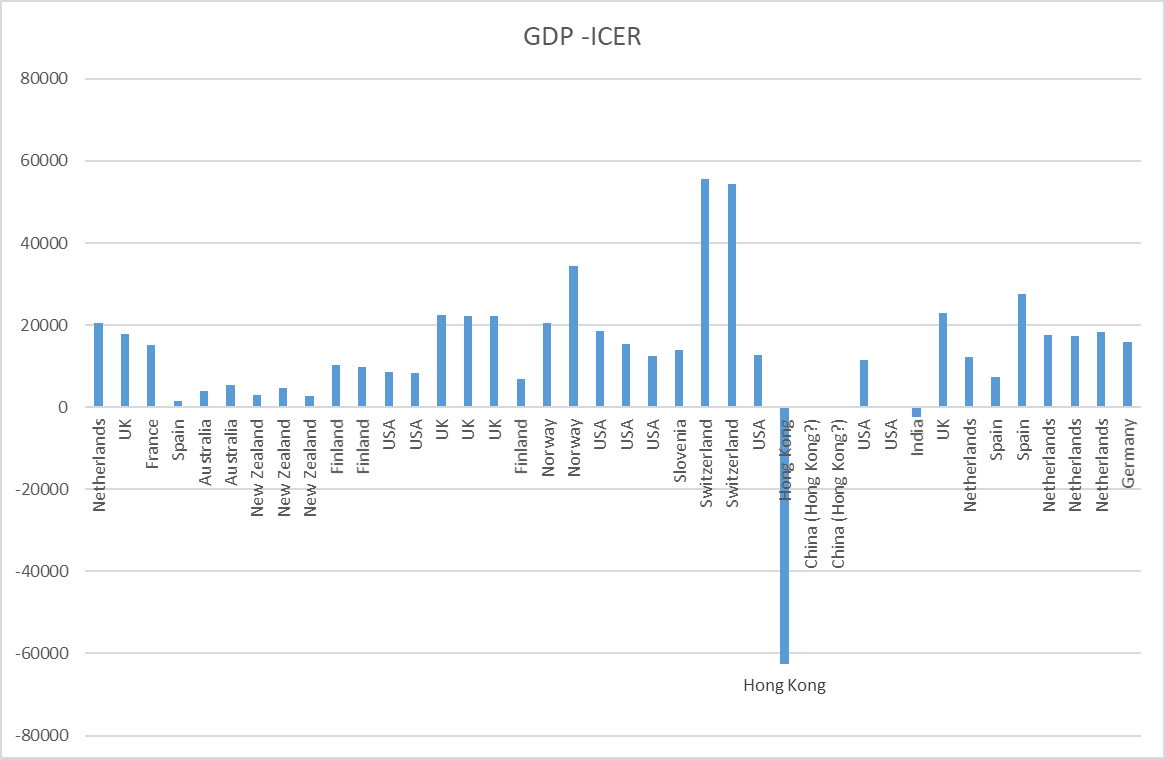


**Appendix 6e. Difference between three gross domestic product per capita and incremental cost-effectiveness ratio (biannual or triennial screening programs for women starting screening at age 50 or above)**

**
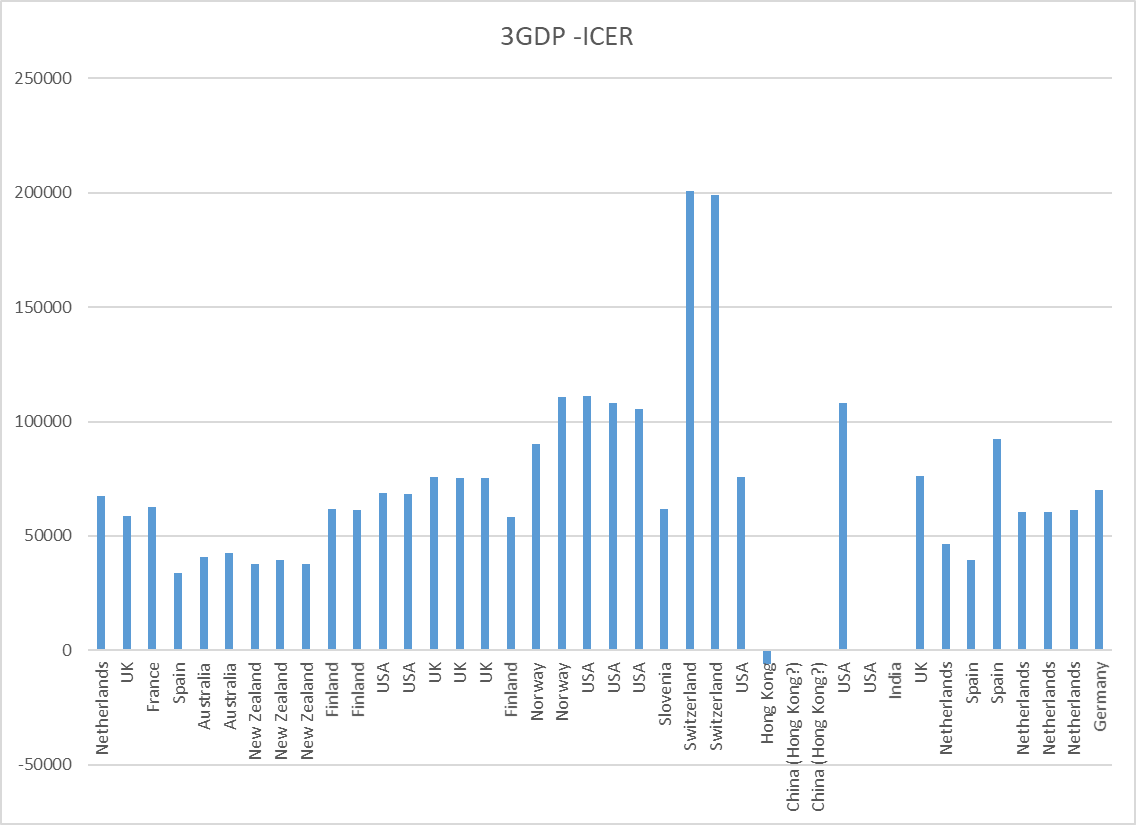
**
